# Analysis of *Posidonia oceanica*’s Stress Factors in the Marine Environment of Tremiti Islands, Italy

**DOI:** 10.3390/molecules29174197

**Published:** 2024-09-04

**Authors:** Martina Fattobene, Elisa Santoni, Raffaele Emanuele Russo, Silvia Zamponi, Paolo Conti, Adelmo Sorci, Muhammad Awais, Fuyong Liu, Mario Berrettoni

**Affiliations:** 1 School of Science and Technology, Chemistry Division, University of Camerino, Via Madonna delle Carceri—ChIP, 62032 Camerino, MC, Italy; martina.fattobene@unicam.it (M.F.); elisa.santoni@unicam.it (E.S.); raffaele.russo@unicam.it (R.E.R.); silvia.zamponi@unicam.it (S.Z.); paolo.conti@unicam.it (P.C.); muhammad.awais@unicam.it (M.A.); fuyong.liu@unicam.it (F.L.); 2 Laboratorio del Ma.Re, Via A. Vespucci, 71040 Isole Tremiti, FG, Italy; info@marlintremiti.it; 3 Henan Collaborative Innovation Center of Environmental Pollution Control and Ecological Restoration, Zhengzhou University of Light Industry, Zhengzhou 450001, China

**Keywords:** *Posidonia oceanica*, metals, BVOCs, oxidative stress, biomonitoring

## Abstract

*Posidonia oceanica* significantly contributes to the health of oceans and coastal areas; however, its progressive decline is becoming an increasing source of concern. The present preliminary study aims to assess the chemical parameters that describe the state of preservation of the aforementioned plant meadows located in the Tremiti Islands archipelago. To better understand the plants’ response to external factors, the emission of biogenic volatile organic compounds (BVOCs) was investigated using *Posidonia oceanica* as a biological indicator. Subsequently, the heavy metal concentrations (Ag, Al, As, Ba, Be, Cd, Co, Cr, Cu, Fe, Mn, Mo, Ni, Pb, Sb, Se, Sn, Ti, Tl, V, Zn) in sediments, leaves, and seawater were determined and pollution indicators were calculated to assess the deviation from the natural background levels of sediments. The dimethyl sulfoniopropionate (DMSP) to dimethyl sulfoxide (DMSO) ratio was calculated to evaluate the oxidative stress levels in the meadows because the DMSP naturally present in *Posidonia oceanica* is oxidized to DMSO and decreases the ratio of DMSP/DMSO. BVOC analysis revealed dimethyl sulphide (DMS) as the most abundant molecule. Morphological features led to variations in metal concentrations across sampling sites, with sheltered bays displaying a higher metal content. Degradation is indicated by a greater DMSO content in the outer leaves. In accordance with the metal content, the bioindicator ratio confirms greater degradation on the south side, which aligns with increased oxidative stress.

## 1. Introduction

*Posidonia oceanica* is an aquatic plant endemic in the Mediterranean Sea where it is found throughout nearly the whole coastline [1]. In the Tremiti archipelago, the plant is likely to be found on the seabed surrounding the San Domino and San Nicola islands. Like all higher plants, *Posidonia oceanica* possesses roots, rhizomes, leaves, flowers, and fruits, as well as ribbon-like leaves that can be 1 or 2 cm wide and up to a metre long. Leaves are organised in bundles with six–seven leaves per bundle and they are arranged alternatingly; therefore, starting from the inside, there is a young leaf, an intermediate leaf, and an adult leaf according to their position, length, and the presence of a lignified base [2].The beam then renews itself from the inside to the outside, experiencing a maximum growth during the spring period [3].

*Posidonia oceanica* can form extensive meadows that can be observed from a depth of 5 to about 30 m. The plants play a crucial role in the coastal marine environment due to their extensive coverage contributing to oxygen production [3] and serving as nurseries and permanent habitats as a support for associated species [3,4]. The meadows contribute to the stabilisation of mobile littoral bottoms through the structure of the ‘matte,’ thereby reducing the impact of currents and waves [2]. Additionally, seagrasses possess characteristics that make them suitable for serving as biological indicators and play a vital role in the global carbon cycle by fixing carbon dioxide from the atmosphere in oceans, making them an essential carbon sink [5].

As a result of the increased urbanisation of coastal areas, the grasslands are unfortunately clearly regressing. The main threats include trawling, free anchoring by recreational boats, presence of port structures, breakwaters, weirs, and artificial canals, all of which disrupt the natural hydrodynamic regime. Additional hazards involve climate change, water eutrophication, sediment anoxia, and diffusive pollution. Furthermore, the destruction of the sedimentation/erosion balance, proliferation of invasive algal species, beach cleaning, increased salinity in the vicinity of desalinators, and pollution by heavy metals pose significant potential risks [1,3,6,7,8].

The most significant disruptions observed within the Tremiti Islands Marine Protected Area (MPA) are consistently found in all the seagrass beds, particularly at their upper boundaries, corresponding to the shallower areas. These areas are adversely affected by numerous anchorages as well as the presence of submerged structures and cables due to the intensive navigation and anchoring activities. Anchoring also causes the resuspension of fine sediment, which leads to a decrease in water transparency. Consequently, these decreases in light exposure, essential for the plant’s life and growth, pose asphyxiation challenges for the seagrass itself. To assess the possible stress factors of *Posidonia oceanica*, three different chemical aspects have been investigated.

The first one was the presence of heavy metals, which is one of the most concerning aspects of environmental contamination in marine waters today; being an aquatic rooted species, *P. oceanica* can absorb elements both through its roots, via sediments, and through its leaves, via the surrounding water. The Mediterranean region is characterised by relatively weak sea currents due to the basin’s structure and shallow depth; consequently, the feeble dispersion of pollutants may exacerbate the accumulation of trace metals along coastlines near contaminant sources and amplify the detrimental effects of toxic substances. The metals analysed were Ag, Al, As, Ba, Be, Cd, Co, Cr, Cu, Fe, Mn, Mo, Ni, Pb, Sb, Se, Sn, Ti, Tl, V, and Zn both on sediments, water, and plants.

Marine sediments are preferred for environmental quality monitoring over seawater because they contain higher and more stable pollutant concentrations. These sediments offer a more comprehensive indication of pollution levels in the area compared to the fluctuating nature of seawater. Moreover, marine sediments serve as both a repository for contaminants and a source of toxicants to marine organisms. To determine potential anthropic contributions, various pollution indicators were calculated, including the contamination factor (Cf), degree of contamination index (Cd), enrichment factor (EF), geoaccumulation index (Igeo), and pollution load index (PLI) for heavy metals and other trace elements [9,10].

The bioconcentration factor (BCF) was a calculated value to assess the ability of plants to remove metals from the soil [11]

The second aspect that has been investigated was the ratio between DMSP and DMSO as a stress indicator [12].

**Scheme 1:**
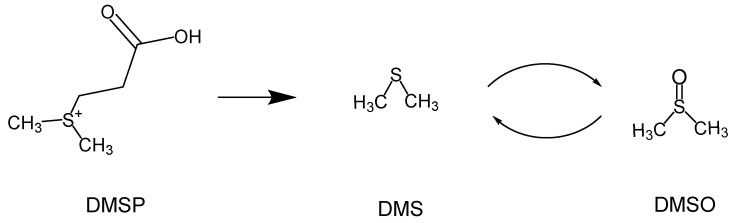
Schematic conversion of DMSP in DMSO and production of DMS starting from those two precursors.

The physiological role of DMSP in *Posidonia oceanica* remains uncertain; however, Refs. [13,14] have suggested many potential features, like herbivore deterrents or antifouling against epiphytes. DMSP and its derivative dimethyl sulfoxide (DMSO) are precursors of dimethyl sulphide (DMS), according to the schematic Scheme 1:

Its production primarily occurs through biogenic processes predominantly stemming from the enzymatic cleavage of DMSP [15]. McFarlin and Alber [16], demonstrated that the DMSP/DMSO ratio can be used as a stress indicator for the higher plant *Spartina alterniflora*, so Champenois & Borges [12], applied this method to *Posidonia oceanica* by analogy obtaining good results, demonstrating that the DMSP/DMSO ratio decreases as environmental stress increases. Richir et al. [17], also studied the weekly, seasonal, and depth variability of the ratio concentration to assess its potential as an indicator of stress, finding out that the ratio remains constant for unstressed plants suggesting its usefulness as an early warning indicator of stress regardless of the season, the depth, or the age of the leaf bundle.

The last aspect considered was the qualitative content of biogenic volatile organic compounds (BVOCs), which are a category of chemicals characterised by low molecular mass (50–200 Da), high vapour pressure under ambient conditions, and from low to moderate hydrophilicity. A wide variety of aquatic organisms produce a diverse range of bioactive compounds, which play a crucial role in ecology and fulfil several purposes including both inter- and intra-specific communication [18,19]. Infochemicals serve as a method of communication, involving molecules that act as messengers within and across species. They induce a behavioural or physiological response in the receiver, which is adaptive for either one or both interacting parties [19]. BVOC production is species-specific, so each plant will favour the production of some compounds over others. In aquatic ecosystems, a variety of biotic and abiotic factors act as triggers that can influence and regulate the production of these metabolites, for example, the cell rupture of microcystis by Daphnia magna leads to the emission of high quantities of β-cyclocitral. Furthermore, the growth of organisms can act as a stressor, in that nutrients and space are being lost, so that the organism’s growth is affected [20,21,22].

The detection of VOCs emitted in response to biotic/abiotic stresses is an important non-invasive tool for monitoring interactions in habitats such as forest and marine communities [19].

This study aims to evaluate the chemical parameters that indicate the preservation state of *Posidonia oceanica* meadows in the Tremiti Island archipelago. This investigation focuses on BVOCs released, the DMSP/DMSO ratio, and the metal content in both the plants and their surrounding environment.

## 2. Results and Discussion

### 2.1. Qualitative Analysis of BVOCs

The qualitative identification of compounds found in the analysed samples by GC-MS was conducted based on the Kovats retention index (KI), using values found in the literature [23], and by comparing the fragmentation pattern of molecules using the NIST17 [24] mass spectrum library. KI was calculated by analysing the alkane mixture under the same analysis conditions as shown in [Table 1].

Because of its inherent formation in marine environments, DMS is abundant at every station. To compare the emission of BVOCs in the inner, intermediate, and outer leaves, radar charts are utilized. GS and CM showed the most significant differences, while P and CS appeared to have similar results (Figures S2.1 and S2.2).

The major compounds present at the Pagliai site are alkanes (5.05% of C17 and 16.86% of C15), aldehydes (5.49% of 2-pentenal and 5.73% of hexanal), and ketones, particularly 1-penten-3-one (7.23%).

**Figure 1:**
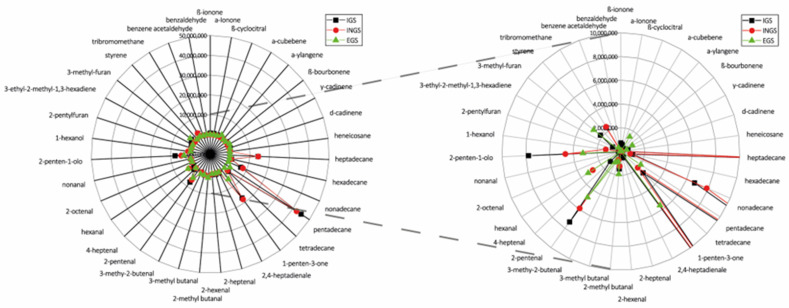
Abundance of different BVOCs in plant analysis of Grotta del Sale station considering the chromatographic peak areas. An enlargement of the central area of the graph is shown on the right-hand side.

At the Grotta del Sale site, there is an abundance of alkanes (9.50% of C17 and 30.15% of C15), aldehydes (4.92% of 2-pentenal), and alcohol (5.22% of 2-penten-1-ol); however, the quantity of terpenes and sesquiterpenes are significantly lower. A peculiarity of this station is the presence of tribromomethane at approximately 1%, distinguishing it from the others. From the radar graph in Figure 1, there is a nearly complete overlap between the inner and intermediate leaves. A small variability is observed in the outer leaves, particularly in the case of α-cubebene and γ-cadinene.

Cala Matano is the only station with an abundant presence of sesquiterpenes in the intermediate and outer leaves, with the major ones being epicubenol (27.46%), isocumene (6.95%), and cadinenes (16.13%). Alkanes are also present, especially in the inner leaves (19.35% of C15), along with carbonyl compounds like 1-penten-3-one (15.59%) and 2-pentenal (5.97%), and alcohol like 2-penten-1-ol (6.55%).

Even at Cala Spido [Figure S2.2], high quantities of alkanes (21.51% of C15, 5.55% of C19, and 6.47% of C17), 2-pentenal (5.55%), and hexanal (4.73%) are observed as aldehydic compounds, along with 2-penten-1-ol (5.75%).

Based on the radar graph [Figure S2.3], it is evident that Cala Matano exhibits a significant variability among intermediate leaves, especially concerning the compounds epicubenol, isocumene, and d-cadinene, while the outer leaves show a good overlap with the inner leaves. However, the inner leaves differ due to an abundance of pentadecane, 2-penten-1-ol, 2-pentenal, and heptadecane.

### 2.2. Metals Content

The analysis of sediments (Figure S3.2) and water (Figure S3.3) employed only samples from the 2nd and 3rd samplings. The standard deviations calculated from the data collected across three replicates at each station indicate relatively low variability between the measurements, with values ranging from 1 to 10%. This suggests good consistency in the data and implies minimal differences between samples, thereby reinforcing the reliability of the measurements. All the reported metal concentrations are referred to the aqua regia-extractable or acid-extractable metal fraction for sediments and water, respectively, and the values are reported in mg/kg (ppm). The values of Ca, K, and Mg ions were excluded from consideration due to their high and coherent content between samplings with mean values around:

- 6300 ppm for Mg, 10,610 ppm for K, and 8000 ppm for Ca in plants [Figure S3.1];
- 2800 ppm for Mg, 3000 ppm for K, and 2300 for Ca in sediments [Figure S3.2];
- 1300 ppm for Mg, 600 ppm for K, and 400 Ca ppm in water [Figure S3.3].

The other metals analysed were Ag, Al, As, Ba, Cd, Co, Cr, Cu, Fe, Mn, Mo, Ni, Pb, Sb, Se, Sn, Ti, V, and Zn. The predominant extractable metal fraction in the specified conditions found in the second sample of water is Ni, with values of about 25 ppm. However, Fe and Cu exhibit greater variability across the sampling sites along with Al and Se, with Cala Spido being identified as the most contaminated location in terms of metal content.

There is a discernible periodic trend observed between the two seawater samplings [Figure S3.4]. Although the Ni content remains comparatively constant, there is a substantial increase in the concentration of Zn. Nevertheless, the quantities of other metals remain constant, consistently identifying Cala Spido as the station with more significant metal-related concerns. This observed pattern may be attributed to the accumulation of pollutants in the area, influenced by the morphological configuration of the cove. Moreover, anthropogenic pollution, likely stemming from the high presence of vessels during the summer months, could be a contributing factor. The unique morphological features of Cala Spido might facilitate the retention of pollutants, leading to an augmented metal content, as indicated by the sustained higher levels of various metals. The significance of ongoing monitoring is highlighted by both natural and anthropogenic factors to assess the evolving dynamics of metal concentrations and to implement effective strategies for the preservation of marine ecosystems.

Due to the fact that Al and Fe emerge as the major constituents [Figure S3.5] of the sediment with values above 2200 and 2400 ppm, respectively, they were analysed separately during the two sampling periods. Furthermore, the decision to analyse them in two distinct sampling periods allows for the assessment of potential temporal variations in their concentrations, resulting in a more comprehensive understanding of the sediment dynamics in the research region [Figure S3.6]. In comparison to Al, Fe content exhibits a decreasing trend between the summer sampling and the one at the end of the tourist season. This trend highlights the significance of considering tourist activity periods as a potential influencing factor in sediment contamination. The identification of this correlation provides a solid foundation for a deeper understanding of contamination dynamics and underscores the importance of considering seasonal factors and human activities in assessing sediment quality.

The extractable metal fraction in the specified conditions in various plant leaves serves as a key indicator for pinpointing critical areas. By combining information from both plant leaves and sediment to gain a first perspective on the dynamics of metal transfer and accumulation in the studied ecosystem, resulting in a more informed and focused environmental assessment. Upon comparing the samples across various sampling sites, there is a noticeable escalation in metal content and consequently, the bioaccumulation in the outer leaves, namely for Fe, Mn, and Zn. Conversely, an opposite trend is observed at the Cala Spido station, indicating a critical situation in the area. The importance of this situation is attributed to a higher concentration of heavy metals such as Ni, Sn, Al, and Pb. The observed increase in metal content, especially in the outer leaves, implies a higher accumulation within the plant tissues. In contrast, the Cala Spido station presents a concerning scenario marked by a reverse trend. The reduced metal content in the outer leaves at this location could signify a potential limitation or alteration in the bioaccumulation process. This is particularly significant in the case of inner leaves, which are expected to be young and less contaminated with heavy metals like Ni, Sn, Al, and Pb. During the second sampling, there is a significant rise in the concentration of all metals, particularly in the inner leaves. This phenomenon may be attributed to a variation in the plant’s metabolism associated with its life cycle, suggesting a potential shift in the plant’s physiological processes. This variation could be linked to distinct phases in the plant’s life cycle, such as growth, development, or response to environmental factors. Plants undergo dynamic metabolic changes as they progress through different life stages, impacting the uptake and distribution of metals within their tissues. The second sampling revealed an increase in metal accumulation in the inner leaves, which highlights the intricate relationship between plant physiology and metal bioaccumulation dynamics. At the Cala Spido and Cala Matano locations, sampling 3 shows a significant variation in the content of Sn and Al, effectively balancing the metal content among the outer, intermediate, and inner leaves. The observed equilibrium in metal content across the various leaf layers in Cala Spido and Cala Matano sites implies a potential spatial influence on the bioaccumulation patterns. The geographical proximity of these two sampling locations may contribute to similar environmental conditions and metal sources, resulting in a more uniform distribution of metals across the different leaf layers. Internal leaves were chosen as a reference to understand bioaccumulation because they are the youngest and healthiest.

**Figure 2:**
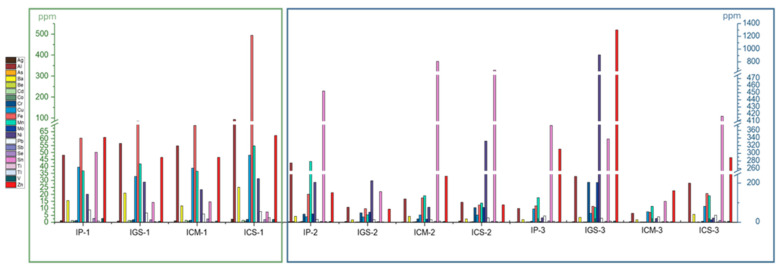
Comparison between internal leaves of 1st, 2nd, and 3rd samplings in each site. The uncondensed version of the image is provided in the Supplementary Materials (Figure S3.7a–c).

From Figure 2 a rising trend in metal content over the three samplings can be observed, particularly for Sn, Zn, and Ni. In a spatial comparison, it is evident that the inner leaves of Cala Spido and Grotta del Sale consistently exhibit higher heavy metal content in all cases. There is an anomaly in the Fe content at Cala Spido in the first sampling, although it remains higher compared to samplings 2 and 3.

### 2.3. Contamination Assessment

The values of background levels reported by Spagnoli et al. and Surricchio et al. [10,25] were used because of the proximity of sampling sites. Be, Mo, Sb, Se, Sn, and Tl were not considered because of the absence of reference values for the background level. Ca, Mg, K, Fe, and Al were not considered because of their high content.

The classification of sediments of [Table 2] was performed following the values reported in [Table 3].

All metals reported low Cf and Igeo values, except for Ti that resulted in very high values and was extremely polluted; Ba was moderately present mainly in Grotta del Sale, Cala Spido, and Cala Matano and was from moderately to strongly polluted.

Sediments resulted in being from high to extremely enriched by almost all metals.

mCd was very or extremely high in all sampling sites, except for the Pagliai station.

All sites resulted in being unpolluted basing on PLI, except for the Grotta del Sale in the 2nd sampling.

Those values confirm that Cala Spido and Cala Matano, being the most enclosed bays, turned out to be the most polluted.

The bioconcentration factors (BCFs) of heavy metals in internal leaves of *Posidonia oceanica* are shown in [Table 4]. Based on this table, a BCF greater than 1 can be found for almost all metals, except Al, As, Fe, Ti, and V, so the plant cannot be considered as a bioaccumulator for these metals. Mo, Ni, Sn, and Zn showed high values even if they are considered as non-essential metals. Ca, Mg, and K were not considered because they are the principal essential nutrients for plants.

### 2.4. Determination of DMSO/DMSP

**Figure 3:**
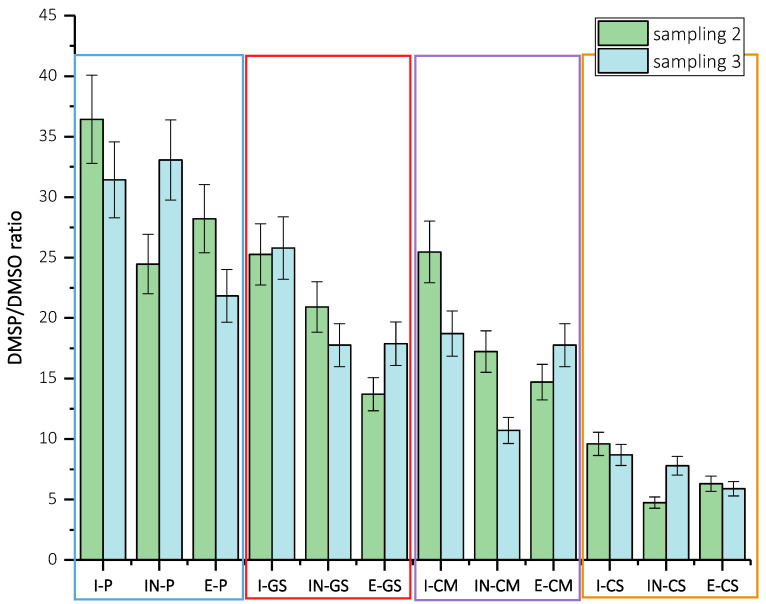
Comparison of DMSP/DMSO ratios in internal (I), intermediate (IN) and external leaves (E) between sampling sites. Error bars indicate the standard deviation calculated on 3 replicates. Each sampling site has been enclosed in a frame: blue—Pagliai, red—Grotta del Sale, purple—Cala Matano, and orange—Cala Spido.

The quantification for the three leaf types was conducted using the relative calibration curves obtained from the DMSO and DMSP standards. The measurement of DMSO and DMSP content is expressed in relation to the weight of each sample, normalised to the fresh weight (fw). The calculation of the ratio between the concentrations of DMSO and DMSP provides an insightful metric for oxidative stress assessment. The values are reported in Table S1.4. The annual growth cycle of the seagrass exhibited a slight fluctuation in DMSO and DMSP content over the two sampling periods with values of DMSO ranging from 0.16 μmol gfw^−1^ to 1.29 μmol gfw^−1^ and DMSP from 4.21 μmol gfw^−1^ to 9.43 μmol gfw^−1^. These values are in accordance with the research conducted by Richir et al. [26], which revealed that DMSP concentrations in *P. oceanica* leaf tissue varied from 20 μmol gfw^−1^ to 265 μmol gfw^−1^ of fresh weight (FW), with DMSO ranging from 2 μmol gfw^−1^ to 5 μmol gfw^−1^. The DMSP:DMSO ratio found by Champenois and Borges [12], ranged from 2 to 40, aligning with the values observed in the examined Posidonia samples. The highest ratio is observed in leaves experiencing lower oxidative stress. A higher ratio is noticeable in all inner leaves compared to the intermediate and outer ones. Additionally, there is a substantial disparity among the four sample sites. More precisely, a significant fluctuation is observed at the Cala Spido site, while a stable trend is noted between the two sampling periods, as shown in Figure 3.

The elevated DMSP/DMSO ratio in leaves with lower oxidative stress is consistent with the existing literature, suggesting that this ratio may serve as an indicator of the plant’s response to environmental stresses. The distinct variations among sampling sites imply potential local influences on the DMSP/DMSO ratio, such as varying environmental conditions or anthropogenic factors. This study reveals that the consistently higher ratio in the inner leaves compared to the intermediate and outer leaves indicates a potential gradient of less stress levels within the plant. This phenomenon could be attributed to the young leaves and factors like nutrient availability, or other site-specific conditions influencing the synthesis or breakdown of DMSP and DMSO. The observed significant variation at the Cala Spido site highlights the sensitivity of *Posidonia oceanica* to local environmental conditions. Having a comprehensive understanding of the specific factors contributing to this variation could provide valuable insights into the plant’s adaptive responses and its role as an indicator of environmental health.

This observation suggests an influence of external environmental factors on DMSP production. The highest DMSP leaf content was observed in the summer, coinciding with the peak of seasonal primary production, especially in shallower depths and younger leaves, where primary production is most prominent. This is consistent with prior studies [14] that suggest the primary production of *P. oceanica* is principally linked to irradiance. It has been demonstrated that increased irradiance causes a rise in the intracellular DMSP content in various photosynthetic organisms, suggesting a possible correlation between the DMSP leaf content of *P. oceanica* and both photosynthesis as well as irradiance.

While a stable trend is noted between the two sampling periods, further investigation is required to explore the reasons behind this stability. It could be attributed to seasonal variations, biological factors, or other dynamic aspects of the plant’s physiology. Overall, the detailed analysis of DMSP/DMSO ratios in *Posidonia oceanica* leaves offers a nuanced understanding of the plant’s stress responses and highlights the complexity of interactions within its ecological context.

## 3. Materials and Methods

### 3.1. Sampling

**Figure 4:**
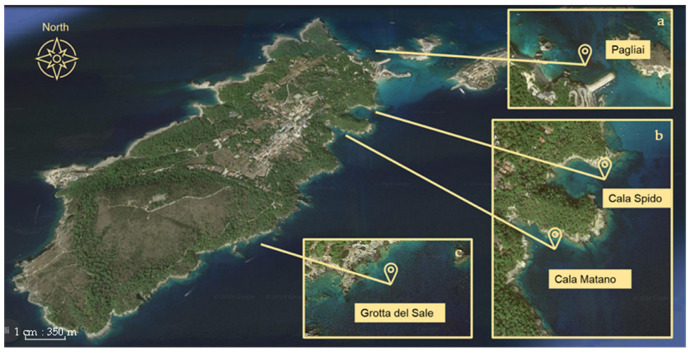
San Domino Island view with zoomed sampling sites (a, b, and c). Darker spots visible in the sea are due to the presence of Posidonia’s meadows.

A series of samplings were carried out along the marine coast of San Domino Island. These sites were chosen after observing a significant yearly variation in the density and health status of *Posidonia oceanica* [27].The chosen sites were identified as Cala Pagliai (P), Cala Matano (CM), Cala Spido (CS), and Grotta del Sale (GS) [Figure 4]. Those four sites are located on the southern area of the island and close to each other. The presence of a shallow sandy seabed allows the growth of *Posidonia oceanica* meadows. The area is subject to fairly high marine traffic due to transport and the large influx of tourists.

Once the sites were identified, the samples were collected based on the analytical method employed. In all analyses, leaves were divided into external (E), intermediate (IN), and internal (I) following the classification used by Richir et al. [26], for the analysis of DMSO/DMSP, and then each analysis was performed using the same categories.

The first sampling (only Posidonia bundles) was obtained during the summer of 2022, while the second and the third samplings took place during the summer of 2023, in July and September, respectively. Only the first sampling was used for BVOC analysis. Additionally, sediments were also collected during the last two samplings [Table 5]. To evaluate the temporal variation in DMSO/DMSP and metal contents, described in Section 3.2, the second and third samples of seawater and *Posidonia oceanica* bundles were used.

Three sampling events were chosen as part of this preliminary study. However, the study design allows for potential future expansions with additional samplings. This approach could facilitate ongoing monitoring and investigations into other chemical parameters, providing a more comprehensive understanding over time.

#### 3.1.1. Water

The water samples (50 mL) for metal analysis were collected at the same depths as *Posidonia oceanica*, using Falcon™ conical tubes, and acidified using 1 mL super-pure HNO_3_ 65% *w*/*w* (Suprapur^®^, Merck, Darmstadt, Germany). Prior to the analysis, samples were filtered using 0.45 µm regenerate cellulose membrane filter from Sartorius (Göttingen, Germany) and diluted with acidified water (1:100 diluted super-pure HNO_3_ 65% *w*/*w*, Suprapur^®^, Merck). According to this procedure the metal contents obtained can be referred to as acid-extractable fractions in the specified condition (~0.3 mol L^−1^ HNO_3_).

#### 3.1.2. Plants

*Posidonia oceanica* bundles were collected and once docked at the harbour, leaves were immediately separated, scraped from epiphytes, and placed in 40 mL vials for the solid phase microextraction (SPME) of fibres with (polytetrafluoroethylene (PTFE) septa. The samples were frozen because the literature [28] showed that this method was optimal for preserving samples for further analyses, instead of drying.

For the analysis of DMSO/DMSP, the samples were removed from the freezer and allowed to dry at room temperature. Leaves were then scraped with a razor blade to effectively remove all the epiphytes, following the method validated by Dauby & Poulicek, [29]. Treated leaves were immediately cut into small pieces and weighted for the analysis. The sampling procedure for the analysis of metals in leaves was conducted using the same procedure employed for BVOCs.

#### 3.1.3. Sediments

Three samples of sediments were collected in proximity of the Posidonia bundle by scuba divers using Falcon™ 50 mL conical tubes directly at the designated sampling point. Once gathered the sediment, the Falcon™ tube was securely sealed to preserve the integrity of the sample and merged before the analytical procedure. This sampling strategy should avoid punctual effects.

### 3.2. Equipment and Analysis

Different analytical procedures were employed for the analysis of BVOCs, metals, and DMSO/DMSP. Quality assurance protocols were applied throughout the experimental phase, using precautionary measures to minimize any systematic errors. The ICP-OES underwent routine calibrations, and GC-FID was utilized to ensure conformity with reference standards. In terms of quality control, data repeatability, reproducibility, and calibration were continually monitored, including cross-verification among operators.

#### 3.2.1. HS-SPME/GC-MS

For the analysis of BVOCs, plant samples were extracted using the SPME method upon reaching room temperature. Divinylbenzene/carboxin/polydimethylsiloxane (DVB/CAR/PDMS, by Supelco) fibre was chosen for the extraction. This fibre has a coating thickness of 50/30 μm, operates within a temperature range of 230–270 °C, and is effective for extracting volatile and semi-volatile compounds, as well as a wide range of aromas and fragrances (PM 20–275). BVOCs were analysed using GC (Agilent GC-4890D, Santa Clara, CA, USA) coupled with an MS detector (Agilent MS-5973N, Santa Clara, CA, USA). The gas chromatograph was equipped with a non-polar column, HP-5MS (30 m × 0.25 mm I.D., 0.10 µm), from Agilent Technology (Santa Clara, CA, USA). The GC temperature ramp starts at 45 °C for 5.5 min, then increases at the rate of 5 °C/min until reaching 170 °C, followed by a further increase at a rate of 20 °C/min until reaching 300 °C, where it is held for 8 min. The mass spectrometer used the Electron Impact (EI) ion source at 70 eV and scan range 29–450 *m*/*z*.

#### 3.2.2. ICP-OES

The quantitative determination of metals was performed using an iCAP™ PRO ICP-OES instrument by ThermoFisher Scientific (Waltham, MA, USA). The quantification was carried out using external standard calibration curve ranging from 1 ppb to 10 ppm. Calibrations were obtained using the MultiElement Standards Ultra Scientific IQC-026 except for P (Sigma Aldrich 207357, St. Louis, MO, USA) and Sn (Merck 43922907, Darmstadt, Germany).

To address potential matrix interferences, particularly from high sodium content in marine water, the evaluation of matrix interference spiking known amounts of sodium into our standards was previously performed. This approach allows the effective assessment and correction of any matrix interferences [30]. The resulting concentrations obtained were consistent with expected values, validating methodology against potential sodium interferences.

LOD and LOQ were automatically computed from the calibration curves by the instrumental software. Samples falling outside the calibration range were appropriately diluted to ensure they fell within the calibration range.

The analysis of marine water sample was conducted after a filtration through a 0.45 µm regenerate cellulose membrane filter from Sartorius and a dilution with acidified water (1:100 diluted super-pure HNO_3_ 65% *w*/*w*, Suprapur^®^, Merck). This dilution was performed to prevent the high concentrations of metals like Na^+^ and Ca^2+^ from interfering with the equipment and to reduce the possibility of interference. In fact, the high salinity of water can pose several challenges in inductively coupled plasma—optical emission spectroscopy (ICP-OES) for which the dilution of samples needed the following:

- Matrix Interference: Elevated concentrations of salts can interfere with the vaporization and ionization of analytes during ICP-OES analysis. This can compromise the precision and accuracy of measurements, as high salt concentrations may alter the composition of the inductively coupled plasma.
- Deposition and Contamination: High salinity may lead to the deposition of salts or other residues inside the instrument, such as in nebulizers and spray chambers. This can cause blockages and contamination that adversely affect the instrument’s performance over time.

To mitigate these issues, it is important to properly treat water samples before analysis. This may involve the filtration and dilution of saline solutions to reduce salt concentrations prior to introduction into the ICP-OES. Subsequently, the analysis was carried out to identify the acid-extractable fraction in the specified conditions

The digestion of sediment samples was performed as follows: approximately 0.5 g of each sample was weighed, followed by the addition of 3 mL of super-pure HCl 36% *w*/*w*, Suprapur^®^ from Sigma Aldrich, and 1 mL of super-pure HNO_3_ 65% *w*/*w*, from Merck, Suprapur^®^ grade (aqua regia). The tubes were then placed inside an ultraWAVE, Milestone Srl digestor, at 230 °C increasing at 8 °C/min and 120 bar for 35 min, following the EPA Method 3051A—microwave-assisted acid digestion of sediments, sludges, soils and oils [31]. After digestion, white residues were observed as the procedure does not dissolve the siliceous part of the sediment. Subsequently, the solutions were brought to a total volume of 50 mL and filtered through a 0.45 µm regenerate cellulose membrane filter from Sartorius before analysis in ICP-OES. For the quantification of the aqua regia-extractable fraction in the specified conditions, all samples were diluted when they did not fall within the calibration curve.

For the analysis of *Posidonia oceanica*, about 100 mg of leaves was put into test tubes, and the digestion was performed in an open-vessel approach using 3 mL of super-pure HNO_3_ 65% *w*/*w*, from Merck, Suprapur^®^ grade. All the tubes were submerged in a sand bath maintained at 80 °C, except for a watch glass covering each tube and the reaction lasted for one hour. Subsequently, hydrogen peroxide (H_2_O_2_ Suprapur^®^ 30% *w*/*w*) by Merck (Darmstadt, Germany) was added to re-oxidize nitrous acid to nitric acid, allowing the reaction to proceed. The temperature was then increased to 100 °C for an additional hour. The solution with aqua regia extractable fraction and without residues was recovered in 25 mL of total volume, using MilliQ water.

#### 3.2.3. HS/GC-FID

The leaves were scraped at room temperature to remove any epiphytes, then cut into small pieces and placed in glass vials (50 mL) equipped with gas-tight PTFE-coated septa. In accordance with the methodology employed by Borges and Champenois [12,14], the DMSP was cleaved into DMS and acrylate by adding 2.5 mL of 6N NaOH purchased from Sigma Aldrich to each vial, which were swiftly sealed thereafter. The reaction was allowed to proceed for at least 24 h at room temperature, even though it was known to be complete within 15 min [32]. This extended duration was chosen to ensure that all the *Posidonia oceanica* cell walls were completely digested. Following the completion of the reaction, a sample of 500 μL was collected using a Hamilton™ 81630 Gastight™ syringe and then manually injected into the GC (AgilentGC-4890D, (Santa Clara, CA, USA)) equipped with FID detector and a non-polar column HP-5 (30 m × 0.32 mm I.D., 0.25 µm) from Agilent Technology (Santa Clara, CA, USA). Subsequently, the septa were pulled out and all the DMS produced by the cleavage of DMSP was removed by gentle stripping with N_2_ gas. Afterwards, 2 mL of hyper-pure HCl 37%, purchased from Sigma Aldrich, was added to acidify the solution and prior to resealing the vial with the coated septa, 1 mL of TiCl_3_, purchased from Merck (Darmstadt, Germany), was also incorporated to initiate the reaction. At room temperature, 2 mL of NaOH was added to remove HCl fumes, and the 500 μL sample was then collected and injected into the GC, using the abovementioned procedure. Quantification was achieved using calibration curves, which were obtained by treating standards of DMSO and DMSP, both purchased from Merck (Darmstadt, Germany), under the same conditions as described in Section S1.

### 3.3. Pollution Indicators

In order to assess the deviation from the natural background levels of sediments, pollution indicators such as the contamination factor (*Cf*), contamination degree (*Cd*), modified contamination degree (*mCd*), enrichment factor (*EF*), the GEOaccumulation index (*Igeo*), and the pollution load index (*PLI*) were calculated [9,10].

The contamination factor (*Cf*) is an index used to evaluate the contamination of a specific substance in a basin, as follows:

(1) $$Cf=\frac{Cs}{Cb}$$

where *Cs* is the concentration of the substance in sediment samples, and *Cb* is the background values for the same element.

The contamination degree (*Cd*) is defined as the sum of all the contamination samples for various heavy metals. The modified one (*mCd*) is related to the number of heavy metals considered (*n*).

(2) $$mCd=\frac{\sum_{1}^{n}Cf}{n}$$

The enrichment factor (*EF*) evaluates the metal contamination and enrichment degree and it normalizes the trace element content with respect to a sample reference metal, such as Al.

(3) EF=MAlsedimentMAlbackground

The GEOaccumulation index (*Igeo*) is an indicator which provides an assessment of heavy metal contamination of sediments with respect to the background natural levels.

(4) $$Igeo={log}_{2}\frac{Cs}{1.5\times Cb}$$

The pollution load index (*PLI*) is an index for the evaluation of contamination status of sediments to heavy metals.

(5) $$PLI=\sqrt[{n}]{Cf1\times Cf2\times \ldots Cfn}$$

The calculation of the bioconcentration factor (*BCF*) was performed as reported in the literature [11].

(6) $$BCF=\frac{Cl}{Cs}$$

where *Cl* is the metal concentration in the leaves.

## 4. Conclusions

*Posidonia oceanica*, an endemic plant of the Mediterranean, is causing increasing concern due to its growing regression particularly in the Marine Protected Area of the Gargano National Park. These factors contribute to defining the main objective of this project, namely, to study possible stress factors to which the plant is subjected through analytical chemical methodologies, analysing their correlation over time and at the four sampling sites.

DMS is the most abundant molecule in leaf-emitted BVOCs, consistently present in all samples along with β-cyclocitral, likely resulting from plant mechanical disruption during sampling. Furthermore, it is worth noting that all samples contained alkanes, aldehydes, ketones, and alcohols. Grotta del Sale stands out for the presence of tribromomethane, whereas Cala Matano exhibits a higher abundance and diversity of sesquiterpenes compared to other stations.

DMS is produced by the cleavage of DMSP, a constituent of leaves that can be transformed into DMSO in the presence of oxidative stress conditions. The ratio DMSP/DMSO as a bioindicator highlighted a greater degradation state on the south side of the island, especially in the Cala Spido and Cala Matano sites with values close to 40. DMSP is more abundant in the younger leaves, consistently associated with photosynthesis and irradiance. The ratio in the inner leaves exhibited a marginal elevation in temporal variability, which might perhaps be attributed to the plant’s metabolic cycle.

Metal analysis revealed higher metal content in sheltered and structurally limited water-circulating bays, like Cala Spido, indicating the distinct morphological characteristics of the sampling sites. Pollutants demonstrate increased concentrations in outer leaves due to prolonged bioaccumulation. Pagliai, Grotta del Sale, and Cala Matano have comparable levels of metal concentrations, which are influenced by the amount of metal present in the sediment at Pagliai. Cala Spido stands out for elevated levels of Sn, Zn, Ni, and Fe, primarily absorbed from the water column and sediments, contributing to an overall higher average metal concentration at this site.

The temporal variation in metal content shows a slightly increasing trend between samplings, especially between the first and second samplings, with a longer time interval.

It is important to note that direct comparisons with other studies can be challenging due to the limited references specific to our sampling site. Furthermore, comparing results from different marine environments may not yield meaningful insights due to varying ecological and geographical factors.

Considering these aspects, it can be hypothesised that the best biological indicator may be the DMSP/DMSO ratio, which identifies Cala Spido as the most vulnerable. It has a higher quantity of metal pollutants, but there is no variation in the emission of BVOCs, which are exclusive to Cala Matano.

From a geographical perspective, it is hypothesized that the two bays, which are only a few hundred meters apart, have a comparable marine environment. Considering the anthropogenic impact due to the maritime traffic at the Tremiti Islands, it is evident that the prairies are dwindling, particularly at Pagliai, where this regression is primarily due to mechanical damage from anchoring despite the fact that the DMSP/DMSO ratio is higher than other sites.

Continuous monitoring of the sites, along with the application of additional analytical techniques for chemical parameters, could help identify further issues at these locations and determine the factors affecting our marine environment.

## Figures and Tables

**Table 1:** Qualitative results of SPME analysis based on the percentage of each compound. KI_C_ Kovats index calculates for HP5 column, KI_N_ Kovats index found in NIST (2017) [24]. KI_A_ Kovats index found in [23].

	Compounds	KI_c_	KI_N_	KI_A_	IP (%)	INP (%)	EP (%)	IGS (%)	INGS (%)	EGS (%)	ICM (%)	INC (%)	ECM (%)	ICS (%)	INCS (%)	ECS (%)
	DMS	-	-	-	32.05	56.39	68.09	13.61	17.44	39.61	24.83	14.76	47.51	20.21	56.25	73.88
Terpenes	β-ionone	1482	1487	1475	0.57	0.41	-	0.49	-	-	0.5	-	-	0.02	0.99	-
	α-Ionone	1423	1428	1430	0.51	0.06	-	0.41	-	-	0.48	-	-	0.03	0.42	-
	β-cyclocitral	1215	1197	1217	0.40	0.08	-	0.22	-	-	0.33	-	0.18	0.06	-	-
Sesquiterpenes	aromadendrene	1429	1441	1441	-	-	-	-	-	-	-	0.36	-	-	-	-
	copaene	1369	-	1376	-	-	-	-	-	-	-	0.20	-	-	-	-
	Cubenene	1525	1541	-	-	-	-	-	-	-	-	2.66	0.19	-	-	-
	cyclosativene	1356	1373	1371	-	-	-	-	-	-	-	0.34	-	-	-	-
	epicubenol	1635	1636	-	-	-	-	-	-	-	-	27.46	2.89	-	-	-
	epizonarene	1519	1528	1501	-	-	-	-	-	-	-	-	0.65	-	-	-
	gleenol	1578	1591	1587	-	-	-	-	-	-	-	2.09	0.13	-	-	-
	isocomene	1383	1393	1387	-	-	-	-	-	-	-	6.95	-	-	-	-
	isoledene	1364	-	1376	-	-	-	-	-	-	-	0.03	-	-	-	-
	laurene	1482	-	1502	-	-	-	-	-	-	-	-	3.69	-	-	-
	sativene	1380	1396	1391	-	-	-	-	-	-	-	0.13	-	-	-	-
	α-cubebene	1344	-	1351	-	0.26	-	0.22	-	2.69	-	3.11	0.35	-	-	-
	α-ylangene	1358	-	1375	-	-	-	-	-	0.07	-	0.02	0.02	-	-	-
	β-bourbonene	1376	1375	1388	-	-	-	0.17	-	0.11	-	0.95	0.10	-	-	-
	β-gurjunene	1420	-	1433	-	-	-	-	-	-	-	0.13	-	-	-	-
	β-ylangene	1409	-	1403	-	-	-	-	-	-	-	0.15	-	-	-	-
	γ-cadinene	1506	1513	1513	-	-	-	-	-	2.04	-	2.01	2.19	-	-	-
	γ-Muurolene	1488	-	1481	-	-	-	-	-	-	-	0.98	-	-	-	-
	δ-cadinene	1518	1527	1523	-	-	-	0.42	-	0.95	-	16.13	0.99	-	-	-
Alkanes	heneicosane	2100	2099	2099	0.06	-	-	0.09	0.08	-	0.02	-	-	0.71	-	-
	heptadecane	1699	1700	1700	5.05	0.50	0.42	9.5	9.56	-	3.99	-	-	6.47	0.68	0.45
	hexadecane	1598	1600	1600	0.31	-	-	0.7	0.55	-	0.39	-	-	0.35	0.04	-
	nonadecane	1900	1900	1900	1.96	-	-	4.53	5.3	-	0.98	-	-	5.55	0.22	-
	pentadecane	1500	1500	1500	16.86	1.6	-	30.15	28.46	3.73	19.35	0.72	1.23	21.51	3.07	2.37
	tetradecane	1398	1400	1400	0.82	0.07	-	1.75	1.32	-	1.17	0.04	0.05	0.88	0.13	-
ketones and Aldehydes	1-penten-3-one	638	684	-	7.23	17.22	7.11	11.64	12.22	10.38	15.59	0.70	5.49	14.03	3.59	-
	2-methyl butanal	652	-	654	1.93	2.13	1.81	0.99	0.84	3.55	1.82	2.14	2.45	1.27	1.51	2.45
	3-methyl butanal	-	-	-	1.12	1.58	1.45	0.58	0.49	1.64	0.93	-	1.38	0.95	1.36	-
	3-methy-2-butenal	782	758	-	-	-	-	-	-	1.9	-	-	-	-	2.31	-
	2-pentenal	741	754	744	5.49	7.73	3.74	4.92	3.98	8.83	5.97	-	5.28	5.55	0.84	-
	hexanal	795	799	801	5.73	6.1	2.14	-	1.91	6.09	3.39	0.08	4.00	4.73	7.56	-
	octanal	994	-	998	-	-	-	-	-	-	-	-	0.27	-	-	-
	3-octen-2-one	1046	-	1030	-	-	-	-	-	-	-	-	1.09	-	0.89	-
	nonanal	1106	1106	1100	-	-	-	0.13	-	-	-	-	0.31	-	0.55	-
	Minor compounds				4.51	-	-	1.81	-	-	2.39	-	0.62	0.11	3.17	-
Alcohols	1-penten-3-olo	633	-	684	1.26	-	-	-	-	-	-	-	-	-	0.95	-
	2-penten-1-olo	759	767	765	5.88	-	-	5.22	3.15	5.92	6.55	-	4.28	3.80	5.75	-
	2,6-dimethyl-cyclohexanol	1110	1103	1102	0.19	-	-	-	-	-	-	-	-	-	-	-
	1-hexanol	866	-	870	-	-	-	-	0.87	-	-	-	-	1.93	1.15	-
OTHERS		1630	-	1627	1.45	0.97	4.84	3.68	1.74	6.29	4.52	9.36	5.35	1.74	3.47	13.15
% of identification					93.4	95.1	89.6	91.2	87.9	93.8	93.2	91.5	90.7	89.9	94.9	92.3
Total area (a.u.)					1.09 × 10^8^	5.45 × 10^7^	4.87 × 10^7^	1.36 × 10^8^	1.30 × 10^8^	5.06 × 10^7^	1.21 × 10^8^	9.57 × 10^7^	8.13 × 10^7^	1.37 × 10^8^	8.81 × 10^7^	2.13 × 10^7^

**Table 2:** Background values, concentration for the elements investigated in sediments, and corresponding pollution indicators, highlighted based on Table 3.

		Sampling 2															
Label	Background	Sediment Pagliai				Sediment Grotta del Sale				Sediment Cala Spido				Sediment Cala Matano			
		SP-2	Cf	Igeo	EF	SGS-2	Cf	Igeo	EF	SCS-2	Cf	Igeo	EF	SCM-2	Cf	Igeo	EF
Ag		<LOD				<LOD				0.8				<LOD			
As	10.0	1.872	0.2	−3.0	30.5	7.5	0.8	−1.0	4.7	3.8	0.4	−2.0	9.9	4.2	0.4	−1.9	14.4
Ba	2.5	9.016	3.6	1.3	587.3	62.5	25.0	4.1	155.9	79.3	31.7	4.4	816.7	20.1	8.1	2.4	278.0
Be		<LOD				<LOD				<LOD				<LOD			
Cd	0.3	0.372	1.2	−0.3	201.9	0.2	0.7	−1.2	4.2	0.3	1.0	−0.6	25.1	0.3	0.8	−0.8	29.0
Co	13.0	<LOD				<LOD				<LOD				<LOD			
Cr	128.0	6.027	0.0	−5.0	7.7	35.2	0.3	−2.4	1.7	14.2	0.1	−3.8	2.9	10.0	0.1	−4.3	2.7
Cu	40.0	12.216	0.3	−2.3	49.7	22.3	0.6	−1.4	3.5	15.2	0.4	−2.0	9.8	13.3	0.3	−2.2	11.4
Mn	774.5	58.504	0.1	−4.3	12.3	238.6	0.3	−2.3	1.9	156.8	0.2	−2.9	5.2	111.9	0.1	−3.4	5.0
Mo		0.946				1.9				0.6				0.2			
Ni	73.0	2.054	0.0	−5.7	4.6	19.1	0.3	−2.5	1.6	6.8	0.1	−4.0	2.4	5.6	0.1	−4.3	2.7
Pb	18.0	4.901	0.3	−2.5	44.3	14.6	0.8	−0.9	5.1	15.8	0.9	−0.8	22.6	10.8	0.6	−1.3	20.8
Sb		0.255				1.0				0.7				0.4			
Se		0.365				<LOD				0.4				0.1			
Sn		0.67				2.0				0.1				0.2			
Ti	0.3	14.746	49.2	5.0	8004.4	125.3	417.7	8.1	2605.5	82.5	275.0	7.5	7085.1	36.7	122.2	6.3	4220.7
Tl		0.03				0.3				0.0				0.1			
V	116.0	5.527	0.0	−5.0	7.8	43.5	0.4	−2.0	2.3	12.0	0.1	−3.9	2.7	11.0	0.1	−4.0	3.3
Zn	48.0	4.787	0.1	−3.9	16.2	42.2	0.9	−0.8	5.5	50.6	1.1	−0.5	27.1	25.4	0.5	−1.5	18.3
		Cd	55.1			Cd	447.5			Cd	310.9			Cd	133.4		
		mCd	4.6			mCd	37.3			mCd	25.9			mCd	11.1		
		PLI	0.3			PLI	1.3			PLI	0.9			PLI	0.6		
		Sampling 3															
Label	Background	Sediment Pagliai				Sediment grotta del sale				Sediment Cala Spido				Sediment Cala Matano			
		SP-3	Cf	Igeo	EF	SGS−3	Cf	Igeo	EF	SCS−3	Cf	Igeo	EF	SCM−3	Cf	Igeo	EF
Ag		0.214				0.2				<LOD				<LOD			
As	10.0	1.353	0.1	−3.5	27.7	2.0	0.2	−2.9	15.4	2.8	0.3	−2.4	15.5	2.7	0.3	−2.5	16.2
Ba	2.5	5.744	2.3	0.6	471.1	14.2	5.7	1.9	426.1	18.6	7.4	2.3	405.3	26.4	10.6	2.8	638.1
Be		<LOD				<LOD				<LOD				<LOD			
Cd	0.3	0.266	0.9	−0.8	181.8	0.2	0.6	−1.3	44.9	0.2	0.6	−1.3	32.3	0.1	0.4	−1.8	25.2
Co	13.0	<LOD				<LOD				<LOD				<LOD			
Cr	128.0	4.417	0.0	−5.4	7.1	5.2	0.0	−5.2	3.1	9.0	0.1	−4.4	3.8	5.7	0.0	−5.1	2.7
Cu	40.0	3.344	0.1	−4.2	17.1	2.3	0.1	−4.7	4.3	3.0	0.1	−4.3	4.0	2.5	0.1	−4.6	3.8
Mn	774.5	62.87	0.1	−4.2	16.6	85.8	0.1	−3.8	8.3	106.9	0.1	−3.4	7.5	91.5	0.1	−3.7	7.1
Mo		0.413				0.4				0.4				0.2			
Ni	73.0	1.513	0.0	−6.2	4.2	2.2	0.0	−5.7	2.2	3.7	0.1	−4.9	2.7	2.8	0.0	−5.3	2.3
Pb	18.0	4.672	0.3	−2.5	53.2	3.0	0.2	−3.2	12.6	3.1	0.2	−3.1	9.5	2.7	0.2	−3.3	9.1
Sb		0.17				0.3				0.4				0.2			
Se		0.389				0.3				0.1				0.2			
Sn		<LOD				<LOD				0.1				0.0			
Ti	0.3	17.205	57.4	5.3	11758.4	26.9	89.5	5.9	6739.0	52.6	175.3	6.9	9541.3	31.3	104.2	6.1	6287.6
Tl		0.064				0.2				0.1				0.1			
V	116.0	4.324	0.0	−5.3	7.6	6.6	0.1	−4.7	4.3	6.8	0.1	−4.7	3.2	6.2	0.1	−4.8	3.2
Zn	48.0	11.462	0.2	−2.7	49.0	14.9	0.3	−2.3	23.4	13.5	0.3	−2.4	15.3	10.1	0.2	−2.8	12.8
		Cd	61.4			Cd	96.8			Cd	184.4			Cd	116.1		
		mCd	5.1			mCd	8.1			mCd	15.4			mCd	9.7		
		PLI	0.2			PLI	0.3			PLI	0.4			PLI	0.3		

**Table 3:** Summary of the classification categories of sediment contamination derived from the different applied indicators.

		**Cf**	Classification
**Igeo**	Classification	<8	low
<0	unpolluted	8–16	moderate
0–1	unpolluted to moderately polluted	16–32	considerable
1–2	moderately polluted	>32	very high
2–3	moderately to strongly polluted	**EF**	Classification
3–4	strongly polluted	≤2	low enrichment
4–5	strongly to extremely strongly polluted	2–5	moderate enrichment
>5	extremely polluted	5–20	high enrichment
**mCd**	Classification	20–40	very high enrichment
<1.5	very low	>40	extreme enrichment
1.5–2	low	**PLI**	Classification
2–4	moderate	<1	unpolluted
4–8	considerable	1–2	unpolluted to moderately polluted
8–16	very high	2–3	moderately polluted
16–32	extremely high	3–4	moderately to highly polluted
>32	ultra-high	4–5	highly polluted
		>5	very highly polluted

**Table 4:** Bioconcentration factors of heavy metals in *P. oceanica* leaves. Values highlighted in green are >1.

BCF Leaf/Sediment	Ag	Al	As	Ba	Cd	Cr	Cu	Fe	Mn	Mo	Ni	Pb	Sb	Se	Sn	Ti	V	Zn
IP-2		0.65	1.73	3.37	3.61	6.77	2.31	0.28	4.76	45.19	99.18	2.91	0.00	10.45	675.09	0.15	0.64	31.46
IGS-2		0.01	0.00	0.21	4.21	1.37	1.22	0.01	0.16	27.29	11.10	0.97	1.15		77.85	0.01	0.01	1.57
ICM-2		0.06	0.07	1.48	3.16	1.73	2.77	0.06	1.21	88.70	13.64	1.23	0.62	59.88	4812.22	0.15	0.30	9.26
ICS-2	7.10	0.04	0.08	0.21	3.64	5.26	2.42	0.03	0.63	129.36	48.51	1.43	0.52	10.68	6250.01	0.03	0.08	1.75
IP-3		0.06	0.00	0.73	8.25	0.52	22.85	0.05	1.17	8.18	6.16	10.36	1.01	57.01	3884.35	0.10	0.13	23.10
IGS-3	13.42	0.69	0.00	4.34	3.96	46.15	13.65	0.15	1.21	491.95	601.64	4.26	3.14	13.49		0.32	1.26	113.53
ICM-3		0.04	0.23	0.48	10.41	0.31	21.22	0.04	0.88	7.61	7.08	10.42	1.14	10.85	4458.29	0.06	0.30	15.83
ICS-3	2.49	0.22	0.00	2.80	7.49	0.85	35.70	0.11	1.58	30.69	9.89	11.72	3.15	29.20		0.18	0.72	19.34

**Table 5:** Localization of each site and condition of sampling (S means south, N means north, E means east) in terms of coordinates, depths, and water temperature. Three samples for each station were taken.

	Coordinates		1st Sampling—22 September 2022		2nd Sampling—16 July 2023		3rd Sampling—1 September 2023	
Performed Analysis			BVOCs, Metals		Metals and DMSO/DMSP		Metals and DMSO/DMSP	
Sample Types			Plant		Plant, Sediment, Water		Plant, Sediment, Water	
Site	Long °E (gg pp ss.dd)	Lat °N (gg pp ss.dd)	Depth (m)	Temperature (°C)	Depth (m)	Temperature (°C)	Depth (m)	Temperature (°C)
Cala Pagliai	15°29′45.0″	42°07′13.0″	13	26.3	12	26.0	13	25.7
Grotta del sale	15°29′29.4″	42°06′32.1″	19	23.0	19	22.4	19	25.4
Cala Matano	15°29′38.8″	42°06′50.7″	16	26.0	17	25.9	14	25.5
Cala Spido	15°29′45.7″	42°06′59.1″	10	27.0	10	27.2	11	25.6

## Data Availability

Data are included in this article/Supplementary Materials/referenced in this article. Other data will be available on request.

## Supplementary Materials

The following supporting information can be downloaded at: https://www.mdpi.com/article/10.3390/molecules29174197/s1, Table S1.1: Preparation of standards by mixing working solution (WS) and NaOH in a total volume of 5.00 mL, Figure S1.1: Calibration curve of DMSO, Figure S1.2: Calibration curve of DMSP, Table S1.2: Calculated LOD and LOQ for DMSO and DMSP, Table S1.3: Relative standard deviation (%) obtained by multiplying SD by 100 and dividing by the mean, Table S1.4: Concentration of DMSO and DMSP for sampling 2 and sampling 3 and relative ratio, Figure S2.1: Abundance of different BVOCs in plant analysis of Pagliai station considering the chromatographic peak areas. An enlargement of the central area of the graph is shown on the right-hand side, Figure S2.2: Abundance of different BVOCs in plant analysis of Cala Spido station considering the chromatographic peak areas. An enlargement of the central area of the graph is shown on the right-hand side, Figure S2.3: Abundance of different BVOCs in plants analysis of Cala Matano station considering the chromatographic peak areas. An enlargement of the central area of the graph is shown on the right-hand side, Figure S3.1: Ca^2+^, Mg^2+^, and K^+^ content in plants, Figure S3.2: Ca^2+^, Mg^2+^, and K^+^ content in sediments, Figure S3.3: Ca^2+^, Mg^2+^, and K^+^ content in water samples, Figure S3.4: Metal content in seawater—comparison between 2nd and 3rd samplings, Figure S3.5: Iron and aluminum content in sediments, Figure S3.6: Metals content in sediments—comparison between 2nd and 3rd samplings, Figure S3.7_a: Internal leaves metal content—1stsampling, Figure S3.7_b: Internal leaves metal content—2ndsampling, Figure S3.7_c: Internal leaves metal content—3rdsampling, Table S3.1: Metal content (ppm) in plant samples; Ca, Mg, K, Tl and Be were not considered and standard deviation (SD) calculated on the three replicates of samples for each site, Table S3.2: Metal content (ppm) in sediment samples and standard deviation (SD) calculated on the three replicates of samples for each site, Table S3.3: Metal content (ppm) in water samples and standard deviation (SD) calculated on the three replicates of samples for each site.
