# Supplementary material for: Analysis of Posidonia oceanica’s Stress Factors in the Marine Environment of Tremiti Islands, Italy
Source: Molecules. 2024 Sep 4;29(17):4197. doi: 10.3390/molecules29174197 (PMC11396999; doi:10.3390/molecules29174197)
Supplement: Supplementary file 1 [file molecules-29-04197-s001.zip › molecules-3000544-supplementary.pdf]

Analysis of *Posidonia Oceanica*'s Stress Factors in the Marine Environment of Tremiti Islands, Italy

Martina Fattobene [1], Elisa Santoni [1], Raffaele Emanuele Russo [1], Silvia Zamponi [1], Paolo Conti [1], Adelmo Sorci [2], Muhammad Awais [1], Fuyong Liu [1,3] and Mario Berrettoni [1,*]

[1] School of Science and Technology, Chemistry Division, University of Camerino, Via Madonna delle
Carceri—ChIP, 62032 Camerino, MC, Italy; martina.fattobene@unicam.it (M.F.); elisa.santoni@unicam.it (E.S.); raffaele.russo@unicam.it (R.E.R.); silvia.zamponi@unicam.it (S.Z.); paolo.conti@unicam.it (P.C.); muhammad.awais@unicam.it (M.A.); fuyong.liu@unicam.it (F.L.)
[2] Laboratorio del Ma.Re, Via A. Vespucci, 71040 Isole Tremiti, FG, Italy; info@marlintremiti.it
[3] Henan Collaborative Innovation Center of Environmental Pollution Control and Ecological Restoration, Zhengzhou University of Light Industry, Zhengzhou 45000, China
* Correspondence: mario.berrettoni@unicam.it

SUPPLEMENTARY MATERIALS

S1. Calibration curves DMSO/DMSP

The calibration curve was obtained by treating the standards in the same way as the samples, so as to not consider the possible incomplete cleavage of the DMSP and DMSO. Calibration curves were constructed using solutions prepared from pure DMSP and pure DMSO. This involved creating a mother solution (SS) with a concentration of 2.04 x 10^{-3} mol/L for DMSP and 2.18 x 10^{-3} mol/L for DMSO, following identical procedures. A working solution (WS) was then prepared with a concentration diluted 1:10 for DMSP and 1:100 for DMSO. The six standard samples were generated by mixing the correct volumes of the two WS, resulting in a total volume of 2.5 ml in vials [Table S1.1.1].

To initiate the cleavage of DMSP into DMS and acrylate, 2.5 ml of NaOH solution (6 mol/L) was added, and the sealed vials were left for 24 hours at room temperature.

Gas samples were collected from the headspace using a gas-tight syringe after the 24-hour period and directly injected into a gas chromatograph (CG) equipped with a flame photometric detector (FPD) for determining DMSP concentration. The capillary column (HP-5) utilized ultrapure He as the carrier gas, and the oven temperature was maintained at 60°C.

Before analysing DMSO, any DMS produced during the cleavage of DMSP was removed by gently bubbling N_2. Subsequently, 2 ml of pure HCl (12 mol/L) was added to acidify the solution and the septa was sealed. Following this, 1 ml of $TiCl_3$ was introduced to reduce DMSO to DMS and the reaction was allowed to proceed for at least 24 hours. Afterward, 1 ml of NaOH (6 mol/L) was injected through the septa to eliminate HCl fumes, and the headspace was equilibrated to atmospheric pressure. Gas samples were then injected into the CG for analysis. It is important to note that in the

blank samples with higher concentrations of DMSP (or DMSO), no DMS peaks were detected, confirming the complete removal of DMS produced from DMSP.

Table S1.1: Preparation of standards by mixing working solution (WS) and NaOH in a total volume of 5.00 ml.

	STD 1	*STD2*	*STD3*	*STD4*	*STD5*	*STD6*
Volume DMSP (ml)	0	0.50	1.00	1.50	2.00	2.50
Volume DMSO (ml)	2.50	2.00	1.50	1.00	0.50	0
Volume NaOH (ml)	2.50	2.50	2.50	2.50	2.50	2.50
Concentration DMSP (mol/L)	0	20.4	40.8	61.2	81.6	10.2
Concentration DMSO (mol/L)	21.9	17.5	13.1	8.8	4.4	0

[Figure S1.1] and [Figure S1.2] show the calibration curves for DMSO and DMSP based on the mixtures of DMSO and DMSP given in [Table S1.1]. The blank for DMSP gave a DMS concentration of zero and the removal of DMS from the DMSP determination prior to the DMSO determination seemed to be complete since the DMSO blank gave zero. It was possible to independently measure DMSP and DMSO in the same vial.

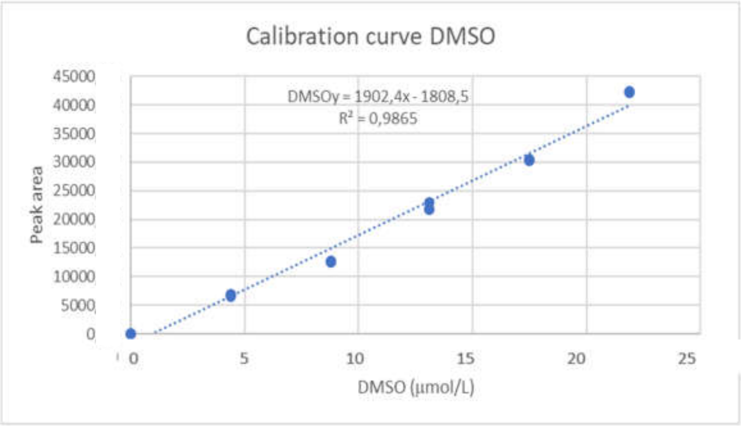

Figure S1.1: Calibration curve of DMSO.

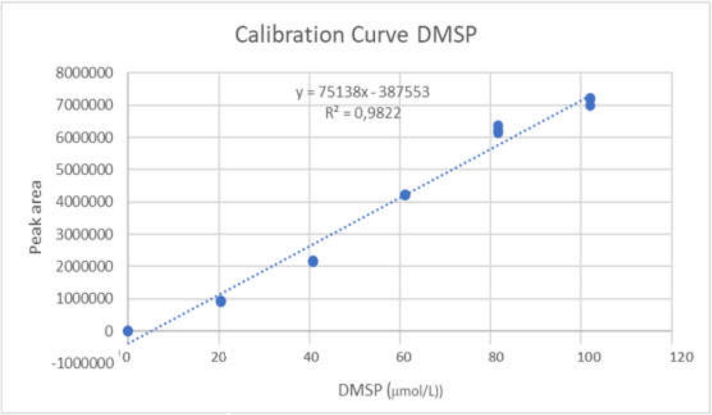

Figure S1.2: Calibration curve of DMSP.

Linearity was performed at different concentrations in the calibration curve range and at lower and higher concentrations to evaluate the ability of our analytical method to give results that are directly proportional to the concentration of the analytes in the samples within a given range of validity. The linearity of multi-point calibration was considered acceptable when R^2 (linear regression square coefficient) ≥ 0.98, S/N (signal to noise ratio)>10, and peaks showed a Gaussian shape. In this case, the coefficient $R^2 \geq 0.98$ and the signal to noise ratio was calculated with GC software from Agilent.

Sensitivity, limit of detection (LOD), and limit of quantification (LOQ) were calculated on the basis of standard deviation of the response and slope of calibration curve, using the following expressions:

$$LOD = 3.3\sigma \,/\, slope \qquad LOQ = 10\sigma \,/\, slope$$

where σ is the standard deviation of the calibration curve at the lowest point of each calibration curve.

Table S1.2: Calculated LOD and LOQ for DMSO and DMSP.

Compound	LOD mg/L	LOQ mg/L
DMSO	0.3120	1.0402
DMSP	0.2189	0.7297

Precision (measurement precision) is a measure of how close results are to one another. One of the values that show precision is relative standard deviation: it is expressed in percent and is obtained by multiplying the standard deviation by 100 and dividing this product by the average [Table S1.3].

Table S1.3: Relative standard deviation (%) obtained by multiplying SD by 100 and dividing by the mean.

**DMSO**	R^2= 0.98651814					**DMSP**	R^2= 0.982182492				
	mmol/L	Peak area	Mean	Dev. std	**RSD %**		mmol/L	Peak area	Mean	Dev. std	**RSD %**
1	0.0	0.00	0	0	**-**	1	0.0	0	0	0	-
1	0.0	0.00				1	0.0	0			
1	0.0	0.00				1	0.0	0			
2	4.4	6.50E+03	6.73E+03	197.8846	**2.94E+00**	2	20.4	9.14E+05	9.21E+05	5.48E+03	**5.96E-01**
2	4.4	6.84E+03				2	20.4	9.24E+05			
2	4.4	6.85E+03				2	20.4	9.24E+05			
3	8.8	1.26E+04	1.26E+04	67.2483	**5.33E-01**	3	40.8	2.16E+06	2.16E+06	7.51E+03	**3.47E-01**
3	8.8	1.26E+04				3	40.8	2.17E+06			
3	8.8	1.27E+04				3	40.8	2.16E+06			
4	13.1	2.17E+04	2.21E+04	655.1369	**2.96E+00**	4	61.2	4.21E+06	4.22E+06	1.79E+03	**4.25E-02**
4	13.1	2.29E+04				4	61.2	4.21E+06			
4	13.1	2.18E+04				4	61.2	4.22E+06			
5	17.5	3.03E+04	3.03E+04	60.61628	**2.00E-01**	5	81.6	6.36E+06	6.24E+06	1.11E+05	**1.78E+00**
5	17.5	3.04E+04				5	81.6	6.14E+06			
5	17.5	3.03E+04				5	81.6	6.21E+06			
6	21.9	4.22E+04	4.22E+04	96.76949	**2.29E-01**	6	102.0	6.99E+06	7.13E+06	1.19E+05	**1.67E+00**
6	21.9	4.21E+04				6	102.0	7.18E+06			
6	21.9	4.23E+04				6	102.0	7.22E+06			

All the DMSO and DMSP sample concentrations are shown in Table S1.4.

Table S1.4: Concentration of DMSO and DMSP for sampling 2 and sampling 3 and relative ratio.

	**SAMPLING 2**							**DMSP/DMSO**	
**ID**	**leaf weight (mg)**	**area DMS**	**conc DMSP**	**μmol/g fw DMSP**	**area DMS**	**conc DMSO**	**μmol/g fw DMSO**	**sampling 2**	**err %**
I-P	1.03E+02	2.50E+04	1.22E+01	1.18E+02	4.13E+05	3.35E-01	3.24E+00	36.44	3.64
IN-P	1.04E+02	1.86E+04	8.84E+00	8.47E+01	4.15E+05	3.61E-01	3.46E+00	24.46	2.45
E-P	1.19E+02	1.98E+04	9.46E+00	7.95E+01	4.13E+05	3.35E-01	2.82E+00	28.22	2.82
I-GS	8.88E+01	1.86E+04	8.83E+00	9.94E+01	4.14E+05	3.50E-01	3.94E+00	25.26	2.53
IN-GS	1.05E+02	2.07E+04	9.94E+00	9.50E+01	4.23E+05	4.75E-01	4.54E+00	20.92	2.09
E-GS	1.19E+02	1.54E+04	7.13E+00	6.00E+01	4.27E+05	5.21E-01	4.38E+00	13.70	1.37
I-CM	5.31E+01	9.82E+03	4.21E+00	7.93E+01	4.00E+05	1.65E-01	3.11E+00	25.46	2.55
IN-CM	1.06E+02	1.33E+04	6.06E+00	5.69E+01	4.14E+05	3.52E-01	3.30E+00	17.23	1.72
E-CM	1.11E+02	1.61E+04	7.52E+00	6.76E+01	4.26E+05	5.11E-01	4.60E+00	14.71	1.47
I-CS	5.56E+01	1.22E+04	5.48E+00	9.87E+01	4.31E+05	5.71E-01	1.03E+01	9.60	0.96
IN-CS	1.07E+02	1.47E+04	6.76E+00	6.31E+01	4.95E+05	1.43E+00	1.33E+01	4.74	0.47
E-CS	1.14E+02	1.83E+04	8.65E+00	7.61E+01	4.91E+05	1.37E+00	1.21E+01	6.30	0.63
	**SAMPLING 3**							**DMSP/DMSO**	
**ID**	**leaf weight (mg)**	**area DMS**	**conc DMSP**	**μmol/g fw DMSP**	**area DMS**	**conc DMSO**	**μmol/g fw DMSO**	**sampling 2**	**err %**
I-P	3.70E+01	9.02E+03	3.79E+00	1.02E+02	3.97E+05	1.21E-01	3.26E+00	31.43	3.14
IN-P	1.11E+02	1.86E+04	8.84E+00	7.95E+01	4.08E+05	2.67E-01	2.40E+00	33.07	3.31
E-P	1.10E+02	1.59E+04	7.41E+00	6.72E+01	4.13E+05	3.39E-01	3.08E+00	21.84	2.18
I-GS	1.03E+02	1.86E+04	8.83E+00	8.56E+01	4.13E+05	3.42E-01	3.32E+00	25.79	2.58
IN-GS	1.01E+02	1.97E+04	9.42E+00	9.36E+01	4.27E+05	5.30E-01	5.27E+00	17.76	1.78
E-GS	1.13E+02	1.04E+04	4.51E+00	4.00E+01	4.07E+05	2.52E-01	2.24E+00	17.88	1.79
I-CM	1.11E+02	1.58E+04	7.37E+00	6.61E+01	4.17E+05	3.94E-01	3.53E+00	18.72	1.87
IN-CM	1.09E+02	1.33E+04	6.06E+00	5.55E+01	4.30E+05	5.66E-01	5.18E+00	10.71	1.07
E-CM	1.07E+02	1.61E+04	7.52E+00	7.03E+01	4.19E+05	4.23E-01	3.96E+00	17.76	1.78
I-CS	2.26E+01	5.24E+03	1.80E+00	8.00E+01	4.03E+05	2.08E-01	9.20E+00	8.69	0.87
IN-CS	1.06E+02	9.67E+03	4.13E+00	3.89E+01	4.27E+05	5.30E-01	5.00E+00	7.79	0.78
E-CS	1.04E+02	1.63E+04	7.60E+00	7.27E+01	4.85E+05	1.29E+00	1.24E+01	5.89	0.59

2. BVOC CONTENT

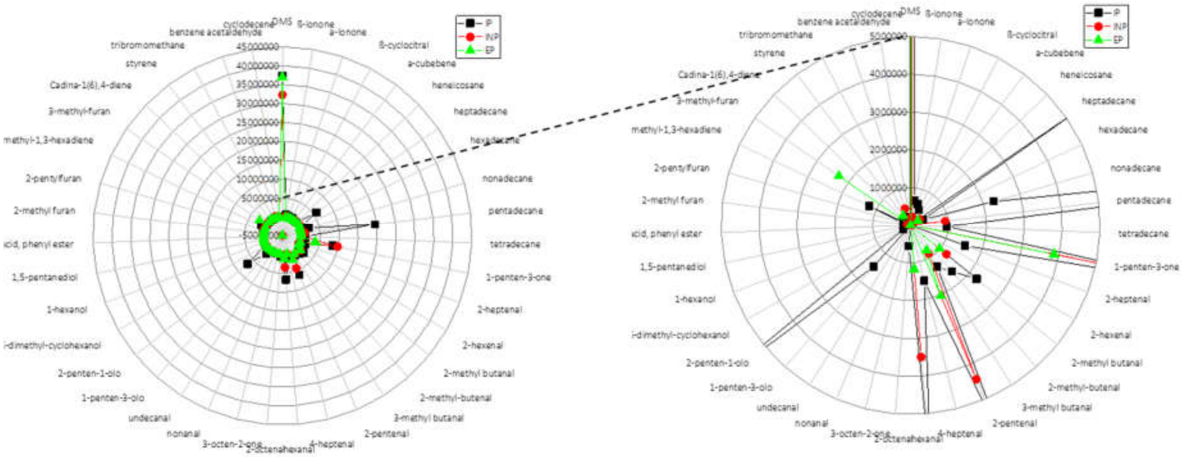

Figure S2.1: Abundance of different BVOCs in plant analysis of Pagliai station considering the chromatographic peak areas. An enlargement of the central area of the graph is shown on the right-hand side.

At the Pagliai site, it was observed that in the inner leaves, the highest variability was attributed to 2-penten-1-ol and hepta-, nona-, and penta-decane. There was a qualitative overlap between the inner and intermediate leaves, while the outer leaves exhibited a lower abundance of emitted compounds and higher variability, particularly in the case of 3-methyl-furan.

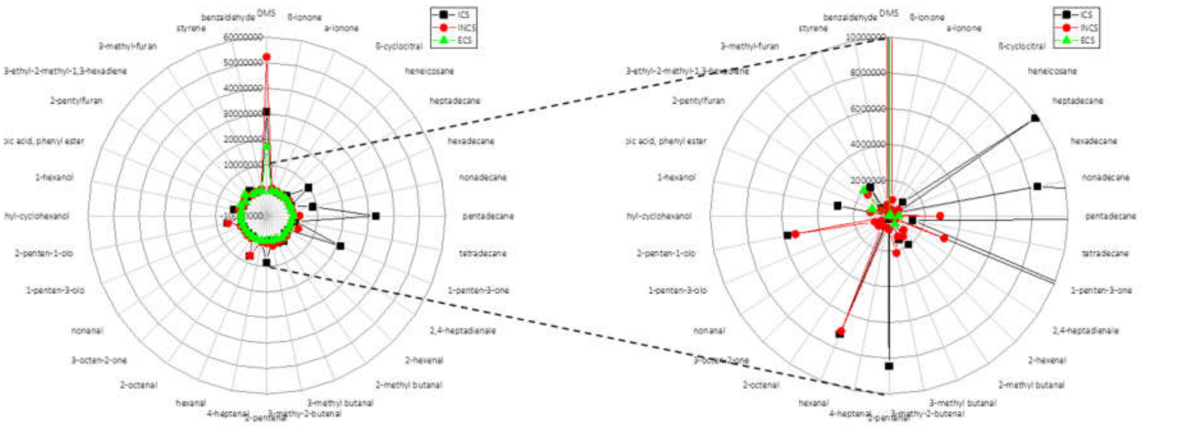

Figure S2.2: Abundance of different BVOCs in plant analysis of Cala Spido station considering the chromatographic peak areas. An enlargement of the central area of the graph is shown on the right-hand side.

Cala Spido exhibits high variability in the inner leaves, attributed to the presence of hepta-, nona-, and penta-decane, 1-penten-3-one, 3-methyl-2-butenal, and 1-hexanol. In contrast, the intermediate and outer leaves show good overlap.

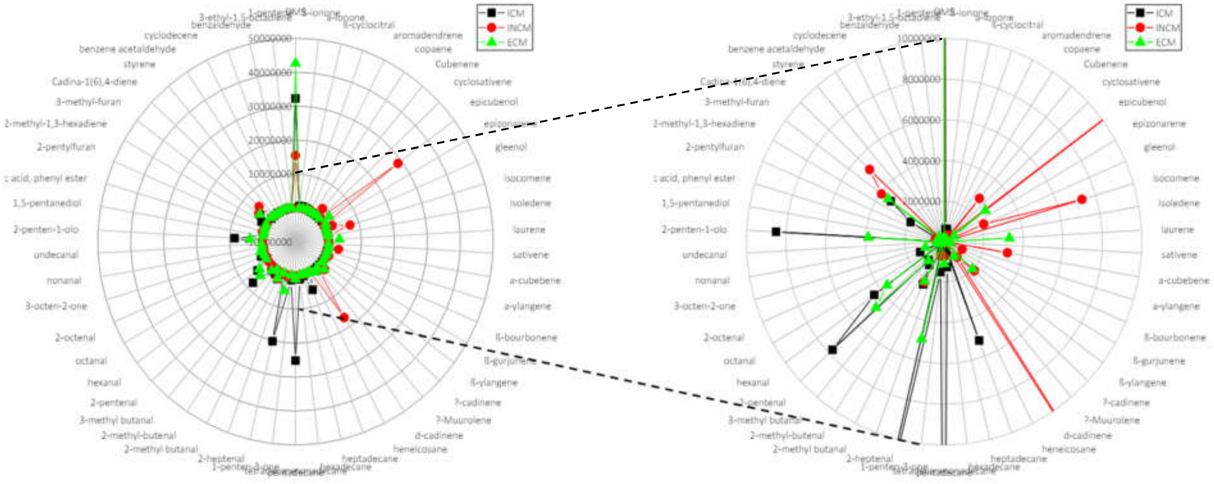

Figure S2.3: Abundance of different BVOCs in plants analysis of Cala Matano station considering the chromatographic peak areas. An enlargement of the central area of the graph is shown on the right-hand side.

Cala Matano exhibits a significant variability in the intermediate leaves, especially concerning the compounds epicubenol, isocumene, and d-cadinene. The outer leaves show a good overlap with both the intermediate and inner leaves. However, the inner leaves differ due to an abundance of pentadecane, 2-penten-1-ol, 2-pentenal, and heptadecane.

S3. METAL CONTENT

The values of Ca, K, and Mg ions were not considered due to their high and coherent content between samplings. Below are the reported graphs with the concentration of those metals in the various samplings [Figure S3.1, Figure S3.2, Figure S3.3].

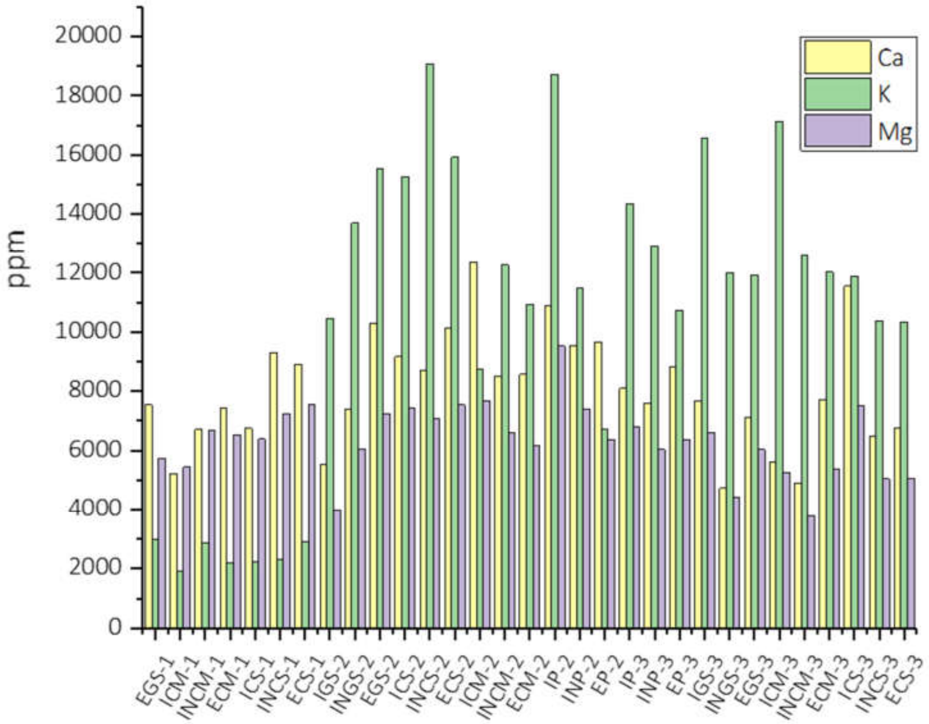

Figure S3.1: Ca^{2+}, Mg^{2+}, and K^{+} content in plants.

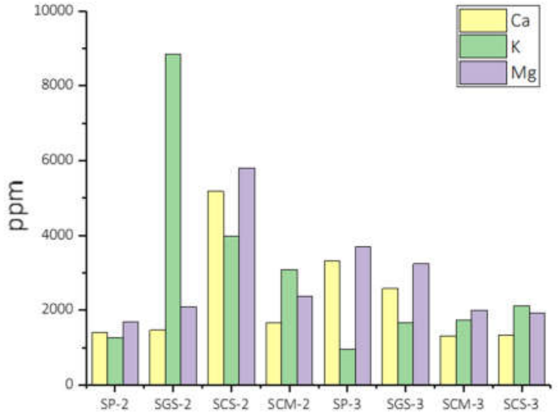

Figure S3.2: Ca^{2+}, Mg^{2+}, and K^{+} content in sediments.

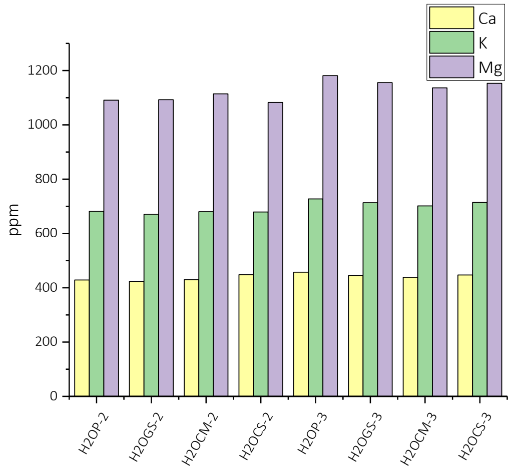

Figure S3.3: Ca^{2+}, Mg^{2+}, and K^{+} content in water samples.

The predominant metal found in the second water samples was nickel; iron and copper, on the other hand, exhibited greater variability across the sampling sites, as aluminium and selenium, with Cala Spido identified as the most contaminated location in terms of metal content.

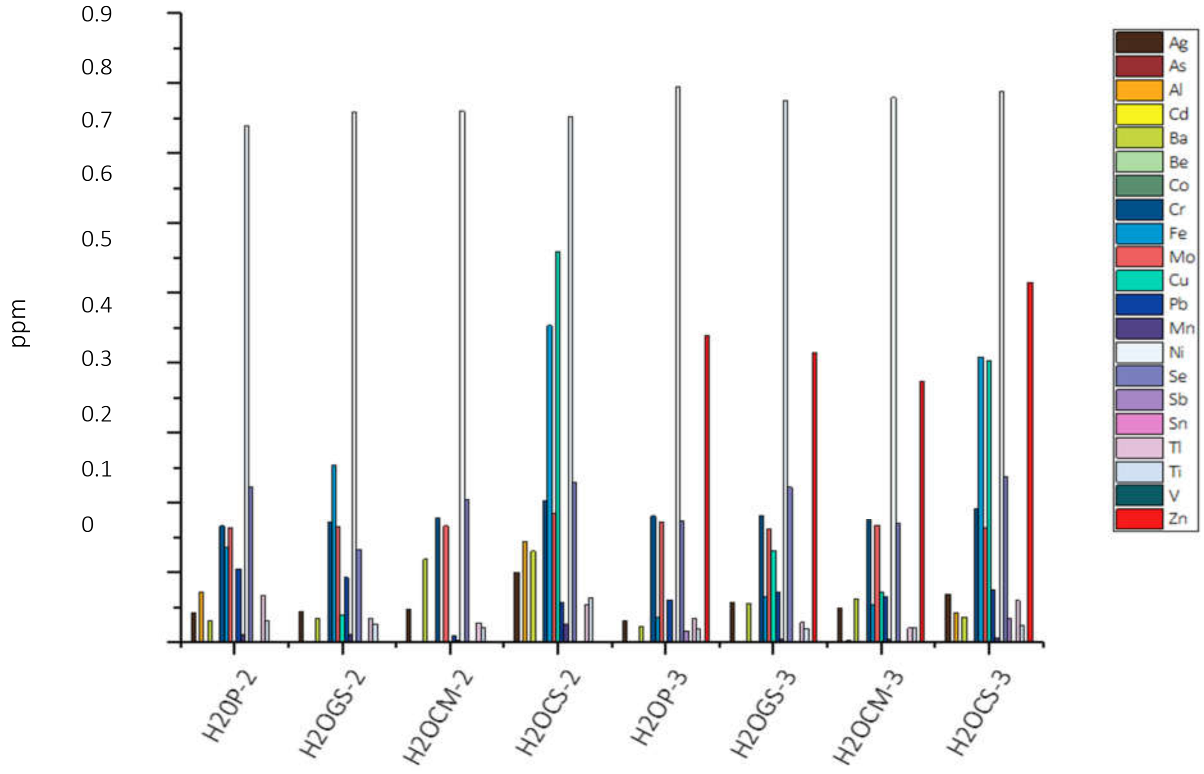

Figure S3.4: Metal content in seawater - comparison between 2nd and 3rd samplings.

The third analysis of seawater consistently revealed patterns across the sampling periods, indicating a notable presence of nickel and zinc. Moreover, there was a discernible upward trend in the concentrations of metals such as copper and lead at the Cala Spido station, further emphasizing it as a location with a higher metal content.

The graph of the seasonal comparisons [

Figure S3.] allows us to discern a periodic trend between the two seawater sampling instances. While the nickel content remains relatively constant, there is a substantial increase in the concentration of zinc. The quantities of other metals, however, remain steady, consistently identifying Cala Spido as the station with greater metal-related concerns. This observed pattern may be attributed to the accumulation of pollutants in the area, influenced by the morphological configuration of the cove. Additionally, anthropogenic pollution, likely stemming from the high presence of vessels during the summer months, could be a contributing factor. The unique morphological features of Cala Spido might facilitate the retention of pollutants, leading to an augmented metal content, as indicated by the sustained higher levels of various metals.

Aluminium (Al) and iron (Fe) emerge as the major constituents of the soil, and as such, they are separately examined in the two sampling periods. The prevalence of aluminium and iron in soil analyses underscores their significance in the composition of the terrestrial substrate. Furthermore, the decision to analyse them in two distinct sampling periods allows for the assessment of potential temporal variations in their concentrations, providing a more comprehensive understanding of the soil dynamics in the study area.

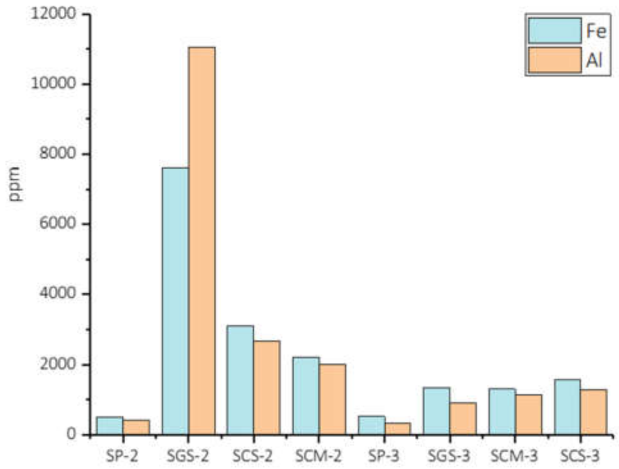

Figure S3.5: Iron and aluminium content in sediments.

From the graph [Figure S3.5], it is evident that iron (Fe) and aluminium (Al) exhibits a decreasing trend between the summer sampling and the one at the end of the tourist season. This trend highlights the significance of considering tourist activity periods as a potential influencing factor in sediment contamination. For instance, during the tourist season, there might be an increase in anthropogenic activities, such as vessel anchoring or the release of pollutants. This could contribute to a gradual depletion of iron content in the sediment. The identification of this correlation provides a solid foundation for a deeper understanding of contamination dynamics and underscores the importance of considering seasonal factors and human activities in assessing sediment quality.

The comparison between the two sediment samplings [Figure S3.6] consistently reflects the concentrations of Fe and Al, designating the initial sampling period as a critical phase for the accumulation of heavy metals. The heavy metals predominantly present include Ti, Zn, and Mn, with the additional detection of Cr, Ni, and As during the initial sampling phase.

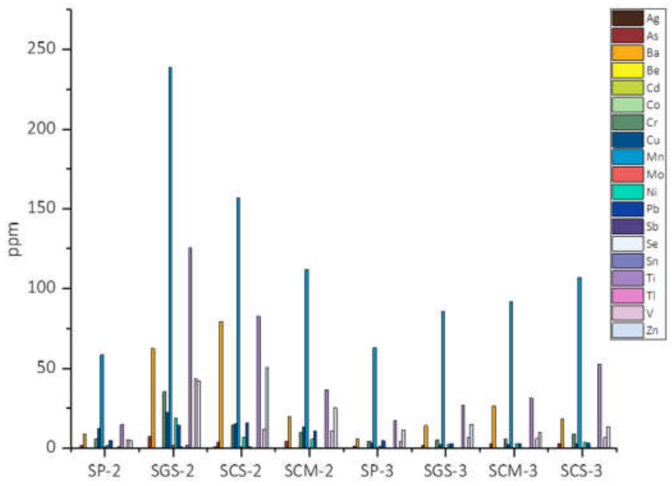

Figure S3.6: Metals content in sediments - comparison between 2nd and 3rd samplings.

This alignment in sediment characteristics emphasizes the significance of the first sampling period as a critical window for heavy metal accumulation. Notably, the presence of heavy metals such as titanium (Ti), zinc (Zn), and manganese (Mn) further underscores the potential environmental impact during this initial phase together with the identification of chromium (Cr), nickel (Ni), and arsenic (As).

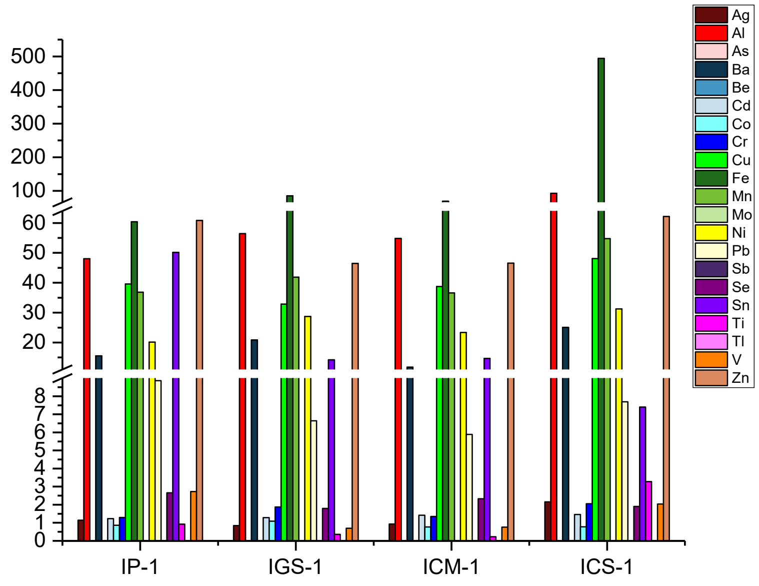

Figure S3.7_a: Internal leaves metal content - 1st sampling

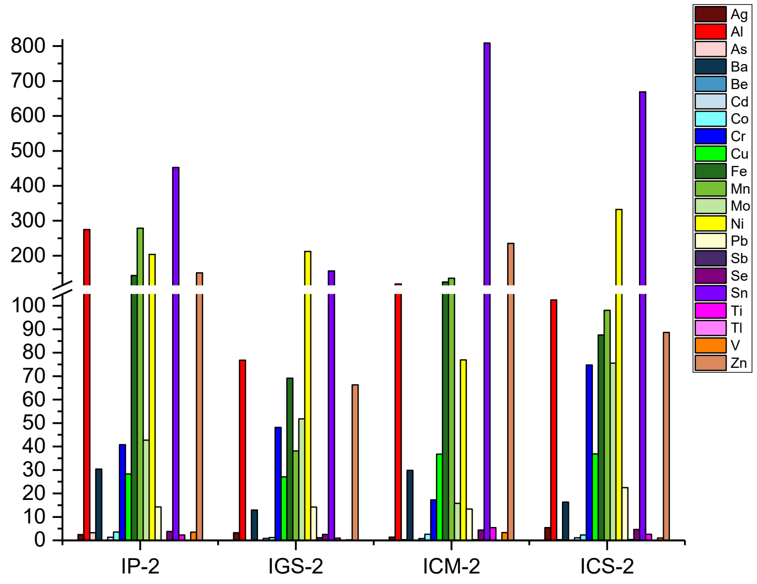

Figure S3.7_b: Internal leaves metal content - 2ndsampling.

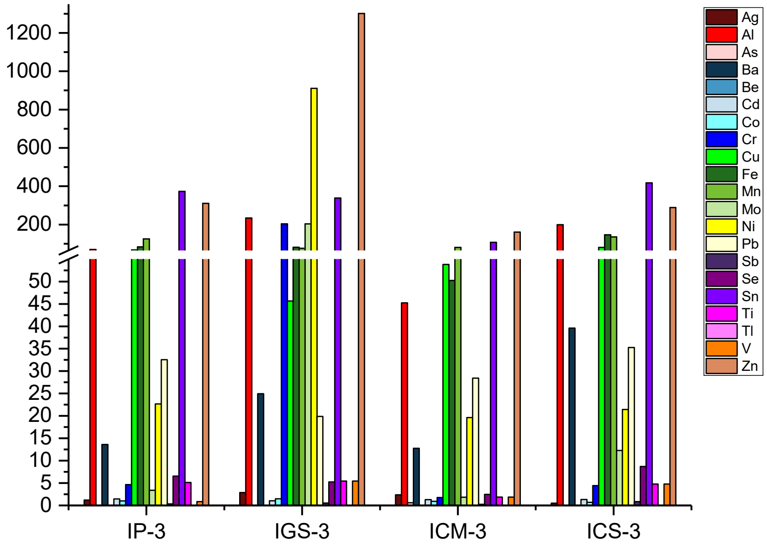

Figure S3.7_c: Internal leaves metal content - 3rdsampling.

Table S3.1: Metal content (ppm) in plant samples; Ca, Mg, K, Tl and Be were not considered and standard deviation (SD) calculated on the three replicates of samples for each site.

	Label	Ag	SD	Al	SD	As	SD	Ba	SD	Cd	SD	Co	SD	Cr	SD	Cu	SD	Fe	SD	Mn	SD
	IP-1	*1.1*	*0.10*	4.8*10^1	*0.9*	<LOD	<LOD	1.5*10^1	*0.9*	1.2	*0.13*	0.86	*0.01*	1.3	*0.10*	3.9*10^1	*2.0*	6.0*10^1	*3.9*	3.6*10^1	*1.6*
	INP-1	*0.88*	*0.12*	4.4*10^1	*2.5*	1.2	*0.10*	1.3*10^1	*0.5*	1.4	*0.10*	2.3	*0.20*	1.5	*0.10*	2.8*10^1	*2.0*	1.8*10^2	*2.1*	1.2*10^2	*1.7*
	EP-1	*0.28*	*0.01*	5.7*10^1	*2.1*	1.3	*0.10*	1.1*10^1	*0.3*	1.2	*0.15*	3.8	*0.30*	1.9	*0.10*	1.8*10^1	*1.8*	1.2*10^2	*8.1*	2.0*10^2	*2.8*
	IGS-1	*0.85*	*0.01*	5.6*10^1	*1.5*	<LOD	<LOD	2.0*10^1	*0.9*	1.3	*0.10*	1.1	*0.10*	1.9	*0.10*	3.2*10^1	*1.7*	8.5*10^1	*8.6*	4.1*10^1	*0.7*
	INGS-1	*0.89*	*0.02*	6.4*10^1	*2.6*	<LOD	<LOD	1.8*10^1	*1.1*	1.4	*0.10*	1.4	*0.01*	4.4	*0.30*	2.8*10^1	*3.4*	1.1*10^2	*11.3*	6.6*10^1	*0.9*
1	EGS-1	*0.48*	*0.01*	7.4*10^1	*4.5*	2.2	*0.10*	1.3*10^1	*0.5*	1.2	*0.16*	2.3	*0.10*	1.3	*0.10*	1.7*10^1	*0.7*	1.6*10^2	*9.9*	1.2*10^2	*2.5*
	ICM-1	*0.93*	*0.02*	5.4*10^1	*2.1*	<LOD	<LOD	1.1*10^1	*0.8*	1.4	*0.10*	0.77	*0.01*	1.4	*0.02*	3.8*10^1	*2.9*	6.9*10^1	*1.3*	3.6*10^1	*0.8*
	INCM-1	*0.58*	*0.01*	1.0*10^2	*5.4*	<LOD	<LOD	1.2*10^1	*1.0*	1.2	*0.10*	2.4	*0.20*	1.6	*0.02*	2.8*10^1	*0.5*	1.6*10^2	*14.1*	1.1*10^2	*3.0*
	ECM-1	*0.36*	*0.01*	9.8*10^1	*2.2*	<LOD	<LOD	1.4*10^1	*1.0*	0.93	*0.12*	2.0	*0.01*	1.4	*0.10*	2.1*10^1	*2.0*	1.8*10^2	*6.2*	1.0*10^2	*51.7*
	ICS-1	*2.2*	*0.40*	9.2*10^1	*5.2*	<LOD	<LOD	2.5*10^1	*2.0*	1.5	*0.10*	0.77	*0.01*	2.1	*0.10*	4.8*10^1	*3.8*	4.9*10^2	*16.3*	5.4*10^1	*2.2*
	INCS-1	*1.4*	*0.30*	1.1*10^2	*4.2*	<LOD	<LOD	1.4*10^1	*0.5*	1.4	*0.10*	2.5	*0.10*	2.3	*0.20*	3.8*10^1	*2.1*	2.7*10^2	*7.9*	1.6*10^2	*4.7*
	ECS-1	*0.85*	*0.04*	9.4*10^1	*7.9*	2.2	*0.22*	1.6*10^1	*0.5*	1.2	*0.01*	4.0	*0.20*	2.7	*0.10*	2.6*10^1	*3.0*	1.7*10^2	*15.8*	2.0*10^2	*15.0*
	IGS-2	*3.3*	*0.10*	7.6*10^1	*6.2*	<LOD	<LOD	1.2*10^1	*0.3*	0.84	*0.02*	1.2	*0.10*	4.8*10^1	*2.71*	2.7*10^1	*1.8*	6.9*10^1	*3.5*	3.8*10^1	*5.7*
	INGS-2	*0.84*	*0.16*	2.9*10^2	*9.9*	2.0	*0.10*	2.2*10^1	*0.9*	1.3	*0.03*	2.4	*0.10*	4.1	*0.21*	1.6*10^1	*0.2*	4.8*10^1	*3.3*	1.5*10^2	*22.8*
	EGS-2	*0.65*	*0.17*	4.7*10^1	*1.5*	5.2	*0.20*	2.3*10^1	*1.4*	2.5	*0.20*	4.4	*0.30*	4.1	*0.33*	1.9*10^1	*1.3*	9.0*10^1	*9.1*	2.6*10^2	*39.1*
	ICS-2	*5.4*	*0.40*	1.0*10^2	*2.8*	0.32	*0.05*	1.6*10^1	*0.3*	1.0	*0.03*	2.3	*0.20*	7.4*10^1	*1.40*	3.6*10^1	*3.7*	8.7*10^1	*4.6*	9.8*10^1	*3.6*
	INCS-2	*1.0*	*0.10*	3.1*10^1	*1.3*	1.7	*0.10*	1.1*10^1	*0.9*	0.87	*0.05*	2.0	*0.10*	1.5*10^1	*0.81*	1.9*10^1	*1.9*	5.4*10^1	*6.4*	1.4*10^2	*24.7*
2	ECS-2	*0.86*	*0.06*	4.9*10^1	*2.9*	4.9	*0.40*	1.2*10^1	*0.8*	1.2	*0.01*	3.0	*0.20*	1.5*10^1	*0.60*	1.3*10^1	*0.8*	8.5*10^1	*3.6*	1.4*10^2	*8.2*
	ICM-2	*1.4*	*0.10*	1.2*10^2	*2.5*	0.31	*0.04*	2.9*10^1	*2.4*	0.80	*0.01*	2.6	*0.10*	1.7*10^1	*0.92*	3.6*10^1	*0.7*	1.2*10^2	*9.2*	1.4*10^2	*2.6*
	INCM-2	*0.63*	*0.03*	1.0*10^2	*8.2*	1.5	*0.10*	1.0*10^1	*0.2*	0.82	*0.04*	1.7	*0.10*	3.7	*0.10*	1.6*10^1	*1.4*	6.5*10^1	*1.2*	1.2*10^2	*10.8*
	ECM-2	*0.80*	*0.08*	4.6*10^1	*3.8*	1.7	*0.10*	9.5	*0.5*	1.1	*0.10*	2.0	*0.10*	1.8*10^1	*1.01*	1.2*10^1	*0.4*	7.7*10^1	*7.2*	1.4*10^2	*4.6*
	IP-2	*2.5*	*0.10*	2.7*10^2	*9.6*	3.2	*0.10*	3.0*10^1	*1.1*	1.3	*0.06*	3.6	*0.10*	4.0*10^1	*1.54*	2.8*10^1	*0.9*	1.4*10^2	*11.2*	2.8*10^2	*9.2*
	INP-2	*0.91*	*0.09*	3.5*10^1	*1.7*	1.9	*0.10*	1.5*10^1	*1.3*	1.0	*0.04*	2.6	*0.20*	1.6*10^1	*0.70*	1.1*10^1	*0.3*	5.8*10^1	*3.2*	1.6*10^2	*4.7*
	EP-2	*0.56*	*0.08*	6.6*10^1	*4.0*	2.3	*0.10*	1.7*10^1	*0.3*	1.2	*0.18*	2.7	*0.10*	3.5	*0.26*	1.1*10^1	*1.0*	1.2*10^2	*13.4*	1.6*10^2	*14.7*
3	IP-3	*1.2*	*0.10*	6.9*10^1	*2.6*	<LOD	<LOD	1.3*10^1	*0.8*	1.5	*0.13*	1.0	*0.10*	4.6	*0.40*	6.7*10^1	*2.6*	8.3*10^1	*7.5*	1.2*10^2	*6.4*
	INP-3	*1.1*	*0.10*	2.7*10^1	*2.0*	0.51	*0.02*	7.3	*0.3*	1.3	*0.18*	2.8	*0.10*	1.5	*0.01*	2.3*10^1	*1.8*	4.8*10^1	*1.9*	2.3*10^2	*15.7*

EP-3	0.20	0.06	5.7*10^1	4.6	1.4	0.10	8.5	0.3	1.0	0.02	2.8	0.20	2.2	0.10	1.7*10^1	1.6	8.2*10^1	8.3	1.8*10^2	18.4
IGS-3	2.9	0.20	2.3*10^2	15.4	<LOD	<LOD	2.4*10^1	1.5	1.1	0.10	1.5	0.01	2.2*10^2	5.54	4.5*10^1	2.3	8.1*10^1	1.6	7.6*10^1	4.0
INGS-3	1.0	0.10	3.1*10^1	2.5	1.1	0.01	1.0*10^1	0.4	0.83	0.03	1.5	0.10	1.1	0.01	2.3*10^1	1.3	3.9*10^1	1.2	1.1*10^2	12.9
EGS-3	1.0	0.01	4.4*10^1	1.7	2.0	0.10	8.4	0.6	0.85	0.02	2.1	0.10	2.3	0.10	1.9*10^1	1.0	7.1*10^1	0.7	1.6*10^2	6.8
ICM-3	2.4	0.20	4.5*10^1	2.4	0.61	0.01	1.2*10^1	1.0	1.3	0.10	0.93	0.10	1.8	0.01	5.3*10^1	4.1	5.0*10^1	2.5	8.1*10^1	6.0
INCM-3	0.71	0.15	3.4*10^1	0.8	0.49	0.01	8.2	0.5	0.78	0.06	1.3	0.10	0.99	0.10	2.1*10^1	0.2	4.1*10^1	3.7	1.0*10^2	1.8
ECM-3	0.26	0.04	9.1*10^1	5.1	1.9	0.10	1.0*10^1	0.8	0.60	0.07	1.5	0.10	1.5	0.10	1.4*10^1	1.0	1.4*10^2	9.0	1.3*10^2	12.3
ICS-3	0.50	0.05	2.0*10^2	7.4	<LOD	<LOD	3.9*10^1	3.3	1.3	0.09	0.68	0.10	4.4	0.27	8.0*10^1	4.4	1.4*10^2	7.3	1.3*10^2	10.6
INCS-3	1.2	0.10	5.5*10^1	2.3	1.2	0.10	1.5*10^1	1.2	1.3	0.10	1.2	0.10	4.2	0.10	3.9*10^1	2.9	6.1*10^1	6.2	2.5*10^2	13.4
ECS-3	0.47	0.05	4.4*10^1	2.0	0.66	0.12	1.3*10^1	0.4	0.88	0.01	2.9	0.20	1.5	0.10	1.9*10^1	0.9	4.9*10^1	0.5	2.0*10^2	22.1

	Label	Mo	SD	Ni	SD	Pb	SD	Sb	SD	Se	SD	Sn	SD	Ti	SD	V	SD	Zn	SD
	IP-1	<LOD	<LOD	2.0*10^1	1.3	8.9	0.9	<LOD	<LOD	2.6	0.2	5.0*10^1	3.0	0.93	0.14	2.7	0.30	6.0*10^1	5.5
	INP-1	<LOD	<LOD	2.2*10^1	0.3	6.4	0.4	<LOD	<LOD	1.2	0.1	3.2*10^1	1.9	1.7	0.10	1.8	0.10	6.5*10^1	3.3
	EP-1	<LOD	<LOD	2.8*10^1	1.9	6.3	0.1	<LOD	<LOD	1.7	0.1	2.9*10^1	0.6	1.0	0.10	1.5	0.10	6.3*10^1	1.3
	IGS-1	<LOD	<LOD	2.9*10^1	2.9	6.6	0.4	<LOD	<LOD	1.8	0.1	1.4*10^1	0.1	0.36	0.02	0.70	0.03	4.6*10^1	0.5
	INGS-1	<LOD	<LOD	3.6*10^1	3.6	6.0	0.6	<LOD	<LOD	2.0	0.1	1.6*10^1	1.1	0.46	0.02	0.86	0.05	4.9*10^1	3.2
1	EGS-1	<LOD	<LOD	2.9*10^1	1.8	9.8	1.0	<LOD	<LOD	1.8	0.0	3.4*10^1	0.4	1.4	0.10	1.4	0.10	4.7*10^1	0.6
	ICM-1	<LOD	<LOD	2.3*10^1	0.4	5.9	0.4	<LOD	<LOD	2.3	0.2	1.5*10^1	1.0	0.23	0.01	0.76	0.02	4.6*10^1	3.1
	INCM-1	<LOD	<LOD	2.4*10^1	2.1	7.2	0.1	<LOD	<LOD	2.1	0.2	4.1*10^1	4.2	1.2	0.10	1.4	0.10	5.9*10^1	5.9
	ECM-1	<LOD	<LOD	1.8*10^1	0.6	5.6	0.5	<LOD	<LOD	1.7	0.2	3.7*10^1	3.7	1.9	0.20	2.0	0.23	3.8*10^1	3.8
	ICS-1	<LOD	<LOD	3.1*10^1	1.0	7.7	0.3	<LOD	<LOD	1.9	0.1	7.4	0.3	3.3	0.40	2.0	0.20	6.2*10^1	2.7
	INCS-1	<LOD	<LOD	2.8*10^1	0.8	8.5	0.9	<LOD	<LOD	2.6	0.1	5.2*10^1	0.7	2.3	0.10	3.6	0.20	5.5*10^1	0.7
	ECS-1	<LOD	<LOD	3.2*10^1	2.9	1.3*10^1	0.3	<LOD	<LOD	3.3	0.2	4.8*10^1	4.8	2.2	0.10	3.1	0.02	8.9*10^1	6.2
	IGS-2	5.1*10^1	1.00	2.1*10^2	12.7	1.4*10^1	0.9	1.1	0.10	2.6	0.1	1.6*10^2	10.2	0.99	0.01	0.3	0.01	6.6*10^1	2.7
	INGS-2	4.2	0.30	4.3*10^1	1.9	8.0	0.6	0.80	0.14	2.1	0.1	4.5*10^1	0.5	0.77	0.02	2.2	0.01	7.1*10^1	3.6
	EGS-2	4.0	0.20	4.9*10^1	0.7	1.1*10^1	0.5	0.91	0.01	2.7	0.2	1.5*10^2	10.1	1.4	0.10	4.3	0.30	8.6*10^1	6.0
2	ICS-2	7.5*10^1	4.50	3.3*10^2	4.5	2.2*10^1	1.5	0.36	0.01	4.7	0.4	6.7*10^2	67.5	2.3	0.04	1.0	0.01	8.9*10^1	2.7
	INCS-2	1.6*10^1	0.30	8.1*10^1	1.3	5.0*10^1	0.6	0.13	0.02	2.3	0.1	1.3*10^2	13.0	0.89	0.10	1.5	0.10	4.9*10^1	2.9
	ECS-2	1.6*10^1	0.20	8.4*10^1	1.2	2.0*10^1	1.3	0.84	0.15	2.5	0.2	1.4*10^2	8.5	1.4	0.10	3.7	0.20	6.2*10^1	3.7

	ICM-2	1.6*10^1	*1.00*	7.6*10^1	*1.6*	1.3*10^1	*1.3*	0.28	*0.01*	4.4	*0.2*	8.1*10^2	*15.4*	5.5	*0.57*	3.3	*0.10*	2.4*10^2	*4.7*
	INCM-2	4.4	*0.10*	3.1*10^1	*0.7*	1.1*10^1	*1.0*	0.68	*0.03*	2.1	*0.2*	1.4*10^2	*5.8*	0.97	*0.05*	1.8	*0.01*	5.6*10^1	*0.6*
	ECM-2	1.6*10^1	*1.10*	8.1*10^1	*2.2*	1.2*10^1	*0.7*	0.18	*0.07*	2.0	*0.1*	1.6*10^2	*11.3*	1.3	*0.01*	4.7	*0.30*	6.0*10^1	*4.0*
	IP-2	4.2*10^1	*4.30*	2.0*10^2	*98.2*	1.4*10^1	*0.3*	<LOD	<LOD	3.8	*0.1*	4.5*10^2	*18.1*	2.3	*0.10*	3.5	*0.01*	1.5*10^2	*1.8*
	INP-2	1.8*10^1	*1.80*	8.9*10^1	*3.6*	7.3	*0.6*	0.31	*0.08*	2.3	*0.2*	1.7*10^2	*5.0*	0.79	*0.01*	3.5	*0.20*	6.7*10^1	*4.5*
	EP-2	3.0	*0.10*	3.0*10^1	*1.3*	1.3*10^1	*0.4*	<LOD	<LOD	2.0	*0.2*	1.3*10^2	*5.1*	1.6	*0.01*	4.4	*0.40*	8.8*10^1	*8.9*
	IP-3	3.4	*0.01*	2.2*10^1	*17.0*	3.2*10^1	*2.0*	0.36	*0.03*	6.6	*0.6*	3.7*10^2	*37.3*	5.1	*0.30*	0.86	*0.10*	3.1*10^2	*30.8*
	INP-3	2.5	*0.01*	2.6*10^1	*4.0*	9.1*10^1	*0.5*	0.32	*0.06*	2.4	*0.1*	1.6*10^2	*10.6*	1.4	*0.01*	1.5	*0.10*	1.4*10^2	*6.0*
	EP-3	2.9	*0.02*	2.3*10^1	*3.7*	9.5*10^1	*0.2*	0.08	*0.04*	2.7	*0.1*	2.1*10^2	*2.6*	1.6	*0.15*	1.6	*0.01*	1.5*10^2	*1.9*
	IGS-3	2.0*10^2	*2.90*	9.1*10^2	*157.6*	2.0*10^1	*0.2*	0.53	*0.09*	5.3	*0.5*	3.4*10^2	*22.6*	5.5	*0.30*	5.5	*0.60*	1.3*10^3	*91.1*
	INGS-3	4.7	*0.19*	1.7*10^1	*0.6*	1.6*10^1	*0.8*	0.06	*0.05*	1.5	*0.1*	1.3*10^2	*13.0*	1.4	*0.01*	1.4	*0.10*	1.0*10^2	*4.1*
	EGS-3	2.7	*0.18*	1.6*10^1	*3.1*	1.6*10^1	*0.2*	0.30	*0.02*	1.9	*0.1*	2.3*10^2	*22.6*	2.0	*0.01*	2.0	*0.10*	1.9*10^2	*9.4*
3	ICM-3	1.8	*0.01*	1.9*10^1	*0.6*	2.8*10^1	*1.9*	0.27	*0.07*	2.5	*0.2*	1.1*10^2	*6.4*	1.9	*0.10*	1.9	*0.02*	1.6*10^2	*11.2*
	INCM-3	7.7	*3.73*	1.8*10^1	*1.6*	1.0*10^1	*1.0*	0.36	*0.09*	1.6	*0.0*	8.8*10^1	*1.7*	1.4	*0.01*	1.4	*0.10*	8.5*10^1	*8.5*
	ECM-3	2.7	*0.10*	1.5*10^1	*1.7*	6.9	*0.7*	<LOD	<LOD	1.8	*0.2*	1.9*10^2	*13.3*	1.8	*0.10*	1.8	*0.20*	7.5*10^1	*4.5*
	ICS-3	1.2*10^1	*0.51*	2.1*10^1	*2.4*	3.5*10^1	*1.6*	0.86	*0.15*	8.7	*0.7*	1.2*10^2	*33.4*	4.8	*0.50*	4.8	*0.40*	2.9*10^2	*5.5*
	INCS-3	4.1	*3.10*	4.4*10^1	*3.1*	1.6*10^1	*0.2*	0.17	*0.02*	1.9	*0.1*	8.0*10^1	*6.4*	1.4	*0.10*	1.4	*0.10*	3.2*10^2	*28.8*
	ECS-3	2.1	*0.30*	2.6*10^1	*1.8*	8.2	*0.6*	<LOD	<LOD	2.0	*0.1*	1.9*10^2	*19.4*	1.0	*0.01*	1.0	*0.03*	1.2*10^2	*10.6*

Table S3.2: Metal content (ppm) in sediment samples and standard deviation (SD) calculated on the three replicates of samples for each site.

**Sampling**	**Label**	***Ag***	***SD***	***Al***	***SD***	***As***	***SD***	***Ba***	***SD***	***Be***	***Cd***	***SD***	***Co***	***Cr***	***SD***	***Cu***	***SD***	***Fe***	***SD***	***Mn***	***SD***
	SP-2	<LOD	<LOD	42.3*10^1	0.043*10^3	1,9	0.1	9.0	0.5	<LOD	0,37	0.01	<LOD	6.0	0.6	1.2*10^1	1.0	0.510*10^3	0.031*10^3	0.58*10^2	0.018*10^2
2	SGS-2	<LOD	<LOD	11.1*10^4	0.696*10^3	7.5	0.7	6.2*10^1	2.1	<LOD	0,20	0.02	<LOD	3.5*10^1	2.1	2.2*10^1	1.3	7.616*10^3	0.259*10^3	2.39*10^2	0.223*10^2
	SCS-2	0.76	0.03	2.68*10^3	0.249*10^3	3.8	0.2	7.9*10^1	1.7	<LOD	0,29	0.01	<LOD	1.4*10^1	0.5	1.5*10^1	0.9	3.111*10^3	0.068*10^3	1.57*10^2	0.092*10^2
	SCM-2	<LOD	<LOD	1.99*10^3	0.119*10^3	4.1	0.5	2.0*10^1	1.7	<LOD	0,25	0.02	<LOD	9.9	0.2	1.3*10^1	1.3	2.216*10^3	0.183*10^3	1.22*10^2	0.063*10^2
	SGS-3	0.22	0.04	9.16*10^2	0.031*10^3	2.0	0.2	1.4*10^1	0.8	<LOD	0,18	0.04	<LOD	5.2	0.4	2.3	0.1	1.339*10^3	0.079*10^3	0.85*10^2	0.083*10^2
3	SCS-3	<LOD	<LOD	1.27*10^3	0.105*10^3	2.8	0.1	1.8*10^1	1.0	<LOD	0,18	0.06	<LOD	8.9	0.5	3.0	0.1	1.575*10^3	0.088*10^3	1.06*10^2	0.024*10^2
	SCM-3	<LOD	<LOD	42.3*10^1	0.067*10^3	2.7	0.3	2.6*10^1	2.6	<LOD	0,13	0.07	<LOD	5.7	0.3	2.5	0.2	1.296*10^3	0.125*10^3	0.92*10^2	0.082*10^2

SP-3	0.21	0.01	3.36*10^2	0.018*10^3	1.6	0.1	5.7	0.5	<LOD	0,27	0.04	<LOD	4.4	0.4	3.3	0.1	0.526*10^3	0.026*10^3	0.63*10^2	0.013*10^2

Sampling	Label	*Ni*	*SD*	*Pb*	*SD*	*Sb*	*SD*	*Se*	*SD*	*Sn*	*SD*	*Ti*	*SD*	*Tl*	*SD*	*V*	*SD*	*Zn*	*SD*
2	SP-2	2,1	0.1	4.9	0.4	0.26	0.03	0.36	0.05	0.67	0.02	1.4*10^1	1.3	<LOD	<LOD	5.5	0.2	4.8	0.5
	SGS-2	19	1.6	1.5*10^1	0.9	0.98	0.16	<LOD	<LOD	2.0	0.20	1.3*10^2	13.1	0.31	0.02	4.3*10^1	2.7	4.2*10^1	4.0
	SCS-2	6,8	0.4	1.6*10^1	0.5	0.70	0.03	0.44	0.06	0.11	0.06	8.2*10^1	5.0	<LOD	<LOD	1.2*10^1	0.4	5.1*10^1	3.0
	SCM-2	5,6	0.3	1.1*10^1	0.2	0.45	0.07	0.07	0.01	0.17	0.04	3.7*10^1	1.2	0.07	0.04	1.1*10^1	0.2	2.5*10^1	0.9
3	SGS-3	2,2	0.2	3.0	0.2	0.27	0.06	0.30	0.02	<LOD	<LOD	2.6*10^1	0.6	0.16	0.05	6.6	0.6	1.5*10^1	0.3
	SCS-3	3,7	0.1	3.1	0.2	0.35	0.06	0.11	0.01	0.09	0.02	5.3*10^1	4.7	0.11	0.04	6.8	0.4	1.3*10^1	1.2
	SCM-3	2,8	0.1	2.7	0.2	0.25	0.08	0.23	0.04	0.02	0.01	3.1*10^1	1.9	0.12	0.01	6.1	0.4	1.0*10^1	0.4
	SP-3	1,5	0.1	4.7	0.5	0.17	0.03	0.39	0.04	<LOD	<LOD	1.7*10^1	1.4	0.06	0.02	4.3	0.1	1.1*10^1	0.8

Table S3.3: Metal content (ppm) in water samples and standard deviation (SD) calculated on the three replicates of samples for each site.

sampling	Label	Ag	SD	As	SD	Al	SD	Cd	SD	Ba	SD	Be	SD	Co	SD	Cr	SD	Fe	SD	Mo	SD	P	SD
1	H2OP-1	0.031	0.010	0.041	0.003	1.9	0.20	0.041	0.003	8.2	0.80	0.010	0.001	0.010	0.001	0.15	0.010	2.9	0.30	0.05	0.01	\	\
	H2OGS-1	<LOD	<LOD	0.020	0.004	0.62	0.01	<LOD	<LOD	0.71	0.01	0.011	0.003	<LOD	<LOD	0.010	0.004	1.2	0.10	0.01	0.02	\	\
	H2OCM-1	<LOD	<LOD	0.032	0.003	0.83	0.02	0.26	0.01	1.7	0.13	0.012	0.002	0.27	0.001	<LOD	<LOD	0.91	0.16	0.03	0.01	\	\
	H20CS-1	<LOD	<LOD	0.021	0.001	0.18	0.03	<LOD	<LOD	0.25	0.04	0.013	0.001	<LOD	<LOD	0.011	0.003	0.89	0.18	0.01	0.01	\	\
2	H20P-2	0.042	0.001	<LOD	<LOD	0.071	0.01	<LOD	<LOD	0.030	0.02	<LOD	<LOD	<LOD	<LOD	0.17	0.010	0.14	0.02	0.16	0.02	8.5	0.7
	H2OGS-2	0.043	0.003	<LOD	<LOD	<LOD	<LOD	<LOD	<LOD	0.031	0.03	<LOD	<LOD	<LOD	<LOD	0.17	0.010	0.25	0.01	0.17	0.03	<LOD	<LOD
	H2OCM-2	0.051	0.004	<LOD	<LOD	<LOD	<LOD	<LOD	<LOD	0.12	0.05	<LOD	<LOD	<LOD	<LOD	0.18	0.010	<LOD	<LOD	0.17	0.02	6.4	0.4
	H2OCS-2	0.10	0.003	<LOD	<LOD	0.14	0.04	<LOD	<LOD	0.13	0.06	<LOD	<LOD	<LOD	<LOD	0.20	0.020	0.45	0.02	0.19	0.03	<LOD	<LOD
3	H2OP-3	0.034	0.001	<LOD	<LOD	<LOD	<LOD	<LOD	<LOD	0.02	0.01	<LOD	<LOD	<LOD	<LOD	0.18	0.010	0.041	0.010	0.17	0.02	<LOD	<LOD
	H2OGS-3	0.061	0.001	<LOD	<LOD	<LOD	<LOD	<LOD	<LOD	0.56	0.08	<LOD	<LOD	<LOD	<LOD	0.18	0.011	0.070	0.020	0.16	0.01	<LOD	<LOD

	H2OCM-3	0.053	0.002	<LOD	<LOD	<LOD	<LOD	<LOD	<LOD	0.62	0.15	<LOD	<LOD	<LOD	<LOD	0.18	0.010	0.052	0.002	0.17	0.02	1.3	0.1
	H2OCS-3	0.070	0.002	<LOD	<LOD	0.043	0.01	<LOD	<LOD	0.04	0.02	<LOD	<LOD	<LOD	<LOD	0.19	0.018	0.41	0.01	0.16	0.01	<LOD	<LOD

sampling	***Label***	***Pb***	***SD***	***Mn***	***SD***	***Ni***	***SD***	***Se***	***SD***	***Sb***	***SD***	***Sn***	***SD***	***Tl***	***SD***	***Ti***	***SD***	***V***	***SD***	***Zn***	***SD***	***Cu***	***SD***
1	H2OP-1	0.33	0.01	0.10	0.01	0.077	0.004	0.038	0.003	0.031	0.003	0.092	0.003	0.012	0.002	\	\	0.044	0.002	3.7	0.3	0.44	0.03
	H2OGS-1	0.048	0.012	0.076	0.003	0.066	0.002	0.010	0.009	0.012	0.002	0.043	0.001	<LOD	<LOD	\	\	0.042	0.004	3.3	0.2	<LOD	<LOD
	H2OCM-1	0.10	0.01	0.29	0.01	0.023	0.001	0.11	0.03	<LOD	<LOD	0.031	0.004	<LOD	<LOD	\	\	0.17	0.01	0.42	0.01	<LOD	<LOD
	H20CS-1	0.015	0.012	0.035	0.003	0.028	0.001	<LOD	<LOD	<LOD	<LOD	<LOD	<LOD	<LOD	<LOD	\	\	0.036	0.005	<LOD	<LOD	<LOD	<LOD
2	H20P-2	0.10	0.01	0.010	0.03	0.74	0.03	0.22	0.05	<LOD	<LOD	<LOD	<LOD	0.073	0.002	0.033	0.003	<LOD	<LOD	<LOD	<LOD	<LOD	<LOD
	H2OGS-2	0.090	0.009	0.014	0.004	0.76	0.05	0.13	0.02	<LOD	<LOD	<LOD	<LOD	0.031	0.003	0.031	0.002	<LOD	<LOD	<LOD	<LOD	0.040	0.009
	H2OCM-2	0.010	0.003	<LOD	<LOD	0.76	0.02	0.22	0.06	<LOD	<LOD	<LOD	<LOD	0.032	0.001	0.023	0.007	<LOD	<LOD	<LOD	<LOD	<LOD	<LOD
	H2OCS-2	0.060	0.001	0.030	0.007	0.75	0.16	0.23	0.05	<LOD	<LOD	<LOD	<LOD	0.051	0.004	0.066	0.003	<LOD	<LOD	<LOD	<LOD	0.56	0.01
3	H2OP-3	0.060	0.003	<LOD	<LOD	0.79	0.12	0.17	0.02	0.025	0.001	<LOD	<LOD	0.034	0.009	0.022	0.002	<LOD	<LOD	0.44	0.04	<LOD	<LOD
	H2OGS-3	0.070	0.001	<LOD	<LOD	0.78	0.01	0.22	0.07	<LOD	<LOD	<LOD	<LOD	0.032	0.003	0.026	0.001	<LOD	<LOD	0.41	0.05	0.13	0.02
	H2OCM-3	0.070	0.003	<LOD	<LOD	0.78	0.05	0.17	0.05	<LOD	<LOD	<LOD	<LOD	0.021	0.001	0.022	0.005	<LOD	<LOD	0.37	0.02	0.070	0.002
	H2OCS-3	0.080	0.004	0.018	0.001	0.79	0.10	0.24	0.02	0.032	0.002	<LOD	<LOD	0.065	0.004	0.024	0.003	<LOD	<LOD	0.52	0.09		

The values reported in Table S3.3, relative to the metal content in water samples, resulted in being slightly higher compared to the analysis carried out by ARPA Puglia along the coast (ARPA Puglia, 2014).
